# Comparative Transcriptome Analysis Reveals New Insight of Alfalfa (*Medicago sativa* L.) Cultivars in Response to Abrupt Freezing Stress

**DOI:** 10.3389/fpls.2022.798118

**Published:** 2022-03-31

**Authors:** Xia Wang, Wenjuan Kang, Fang Wu, Jiamin Miao, Shangli Shi

**Affiliations:** College of Grassland Science, Gansu Agricultural University, Lanzhou, China

**Keywords:** *Medicago sativa*, freezing stress, transcriptome, ABC gene family, Ca^2+^ signal transduction, CBFs/DREB1s

## Abstract

Freezing stress is a major limiting environmental factor that affects the productivity and distribution of alfalfa (*Medicago sativa* L.). There is growing evidence that enhancing freezing tolerance through resistance-related genes is one of the most efficient methods for solving this problem, whereas little is known about the complex regulatory mechanism of freezing stress. Herein, we performed transcriptome profiling of the leaves from two genotypes of alfalfa, freezing tolerance “Gannong NO.3” and freezing-sensitive “WL326GZ” exposure to −10°C to investigate which resistance-related genes could improve the freezing tolerance. Our results showed that a total of 121,366 genes were identified, and there were 7,245 differentially expressed genes (DEGs) between the control and treated leaves. In particular, the DEGs in “Gannong NO.3” were mainly enriched in the metabolic pathways and biosynthesis of secondary metabolites, and most of the DEGs in “WL326GZ” were enriched in the metabolic pathways, the biosynthesis of secondary metabolites, and plant-pathogen interactions. Moreover, the weighted gene co-expression network analysis (WGCNA) showed that ATP-binding cassette (ABC) C subfamily genes were strongly impacted by freezing stress, indicating that *ABCC8* and *ABCC3* are critical to develop the freezing tolerance. Moreover, our data revealed that numerous Ca^2+^ signal transduction and CBF/DREB1 pathway-related genes were severely impacted by the freezing resistance, which is believed to alleviate the damage caused by freezing stress. Altogether, these findings contribute the comprehensive information to understand the molecular mechanism of alfalfa adaptation to freezing stress and further provide functional candidate genes that can adapt to abiotic stress.

## Introduction

Alfalfa (*Medicago sativa* L.) is one of the most important cultivated perennial forage legume species in the world (Yang et al., 2010), and it is grown extensively in different temperature zones based on its high biomass yield, rich nutritional value, good palatability, high capacity for nitrogen fixation, and strong ecological adaptability (Chao et al., 2019). However, under extreme environmental conditions, freezing stress is a major factor that substantially attenuates alfalfa growth, development, productivity, and distribution (Song et al., 2016; Zhou et al., 2018; Castonguay et al., 2020), especially in northern cold regions (Anower et al., 2016). Moreover, unusual abrupt temperature changes lead to serious economic losses in the winter and later spring frost events (Zhou et al., 2018). Therefore, clarifying the mechanisms responsible for freezing tolerance during periods of abrupt freezing stress is important for the breeding of novel alfalfa varieties.

Freezing stress (below 0°C) initially causes ice formation in the cell wall, which directly affects cellular metabolic activities. However, as the ice crystals grow, water uptake by the call is reduced leading to cellular acute dehydration and severe damage to the cell membrane (Ahmed et al., 2015). Oxidative stress severely affects the activities of enzymes in plants, such as reactive oxygen species (ROS) scavenging system enzymes. Furthermore, freezing stress induces instability of protein complexes and RNA secondary structures in cell physiology. Finally, plants perish because freezing stress damages the photoinhibition and destroys the metabolic balance in plants (Gong et al., 2020). To adapt to freezing stress, plants have evolved a series of strategies to increase their freezing tolerance (Tsutsui et al., 2009). However, the freezing tolerance level is a diversiform change in physiological, biochemical, molecular, and morphological characteristics in different plant species (Eremina et al., 2016). ROS are important biochemical changes that severely affect plant growth. Plant cells scavenge ROS to induce related systems, including superoxide dismutase (SOD), catalase (CAT), peroxidase (POD), and ascorbate peroxidase (APX; Karimzadeh et al., 2021). Enhanced antioxidant activities increase freezing tolerance in many plants, such as maize (*Zea mays* L.; Hodges et al., 1997), rice (*Oryza sativa* L.; Huang and Guo, 2005), chickpeas (*Cicer arietinum* L.; Kaur et al., 2009), and alfalfa (Wang et al., 2009).

In addition, freezing stress can trigger rapid increase in calcium (Ca^2+^) in the cytosol (Gong et al., 2020). Ca^2+^, as a second messenger, strongly affects primary low-temperature stress signal transduction (Monroy and Dhindsa, 1995) and decoded by downstream effector proteins, including calmodulins (CaMs), CaM-like proteins (CMLs), calcium-dependent protein kinases (CDPKs), and calcineurin B-like proteins (CBLs), to generate related responses to stress (Zhu et al., 2015). CaM is present in all intracellular events in plants; it is a ubiquitous Ca^2+^ sensor protein. Moreover, CaMs interact with more than 300 target proteins and modulate their activities to defend against environmental stress (Yang and Poovaiah, 2003). Recent studies have demonstrated that many CaMs/CML genes are induced by freezing stress to improve the freezing tolerance, such as *NpCaM-1* (Zeng et al., 2015), *AtCaM3* (Townley and Knight, 2002), *AtCML24* (Delk et al., 2005), *AtCML10* (Cho et al., 2016), *CsCML16*, *CsCML42* (Ma et al., 2019), *MtCML24*, and *ShCML44* (Sun et al., 2021). In contrast, CDPKs directly modulate Ca^2+^ signals at the cytosolic level (Mall et al., 2011) to involved in resistance to abiotic stresses, including cold stress, and many CDPK-related genes can be induced under freezing stress to adapt to changes in temperature (Komatsu et al., 2007; Ray et al., 2007; Dubrovina et al., 2015). However, the mechanisms of CDPK-positive or negative regulated resistance to freezing stress are not clear. In *Populus euphratica, PeCPK10* positively regulates freezing tolerance (Chen et al., 2013). However, *ZmCPK1* in maize negatively regulates cold tolerance through the Ca^2+^ signaling pathways (Weckwerth et al., 2015). Similar to the CBL genes, multiple CBL genes conferring stress have been identified in different plant species (Kolukisaoglu et al., 2004; Li et al., 2012; Sun et al., 2015; Zhang et al., 2016; Wang et al., 2020). Several genes related to CBLs, such as *BrCBL1* (Lee et al., 2013), *PeCBL6*, and *PeCBL10* are upregulated in response to cold stress (Li et al., 2012) and can increase *CBF3*, *COR15A*, and *COR47A* expression levels to confer freezing stress (Zhou et al., 2016). However, Cheong et al. (2003) reported that *AtCBL1* is downregulated under cold stress inhibiting the expression of *AtCBF3* to cope with environmental changes. In addition to Ca^2+^ signal transduction, which is important in freezing stress, the mitogen-activated protein kinase (MPK) cascades and phytohormones also play a key role in the freezing signal process (Li et al., 2017; Liu et al., 2020). To mitigate freezing stress, several transcription factors are activated, regulating the expression of downstream cold-regulated (COR) genes (Peng et al., 2021).

CBF/DREB1 (C-repeat binding factors/Dehydration responsive element binding protein 1) is an important signal transduction pathway involved in freezing tolerance mechanism. CBFs, including CBF1, CBF2, and CBF3, are the AP2/ERF family transcription factors, which regulate COR genes to confer freezing stress. Accordingly, overexpressed CBFs increase the freezing tolerance in diverse plant species (Song et al., 2016). In Arabidopsis, *CBF1* and *CBF3* positively regulate freezing tolerance (Tsutsui et al., 2009), and overexpressing *MtCBF3* could enhance freezing tolerance in *Medicago truncatula* (Tayeh et al., 2013). Moreover, Shu et al. (2017) observed nine CBF unigenes of alfalfa homology at the *Mt-FTQTL6* site, which positively regulated freezing tolerance. Additionally, Kanchupati et al. (2017) suggested that CBFs may strongly impact the freezing tolerance of alfalfa. However, because the alfalfa genome has not been fully sequenced, the molecular mechanism of CBF cluster responses to freezing stress is not clear.

Recently, with the development of high-throughput RNA-sequencing (RNA-seq) technology applications, the mechanisms of freezing stress response in numerous plants have been revealed, and thousands of freezing tolerance-related genes are involved in signal transduction. These studies suggest that transcription factors regulate gene expression to defend against freezing stress (Nah et al., 2016). However, little is known about the response to abrupt freezing stress in alfalfa (Song et al., 2016). In this study, we utilized RNA-seq to evaluate changes in freezing stress response genes in leaves of “Gannong NO.3” (freezing tolerance) and “WL326GZ” (freezing sensitive) with abrupt freezing stress based on the referenced alfalfa genome (Chen et al., 2020), and identified several genes that may strongly impact the freezing stress response in alfalfa. These results elucidate the mechanism of freezing stress response and can be used to compare the freezing tolerance and freezing sensitivity in alfalfa. This study will provide valuable resources for practitioners performing freezing tolerance molecular breeding in alfalfa.

## Materials and Methods

### Plant Materials and Culture Conditions

Two freezing tolerance alfalfa cultivars were studied. The first was the freezing-tolerance cultivar “Gannong NO.3” (fall dormancy score of 3.0), which is produced in Gansu, Ningxia, Xinjiang, and eastern margin of the Qinghai-Tibet plateau. Its seeds were provided by Gansu Agricultural University. The second was the freezing-sensitive “WL326GZ” (fall dormancy score 3.8), which was purchased from Zhengdao Ecological Technology Co. (Beijing, China). All seeds were sown on arenaceous quartz in a growth room, and each sample with three times. The growth conditions were as follows: 200 μmol m^−2^ s^−1^, a light period of 14 h at 20°C, with 10 h dark at 18°C, and humidity ranging from 60 to 80%. The seedlings were irrigated with half-strength Hoagland solution daily after the seeds germinated. Four weeks later, the seedlings were transferred to a cold chamber at −10°C. All leaves were harvested at 0, 0.5, 1, and 2 h after freezing stress. Detailed sample names are as follows: G01-03, G11-13, G21-23, and G31-33 represent three “Gannong NO.3” samples harvested at 0, 0.5, 1, and 2 h, respectively; W01-03, W11-13, W21-23, and W31-33 represent three “WL326GZ” samples harvested at 0, 0.5, 1, and 2 h, respectively. Thus, every sample had three replicates, and the leaves were frozen in liquid nitrogen and stored at −80°C until use.

### Enzyme Extraction and Assays

To investigate the contents of four types of antioxidant activities (superoxide-SOD, peroxidase-POD, ascorbate peroxidase-APX, and catalase-CAT), we used 0.1 g fresh leaves which were homogenized in 1.5 ml potassium phosphate buffer (50 mM, pH 7.0) and centrifuged for 20 min at 12,000 r min^−1^with 4°C for obtaining the upper supernatant to detect the antioxidant activities. The SOD activity was measured according to method reported by Giannopolitis et al. (1977), POD activity was determined using the guaiacol oxidation (Chance and Maehly, 1955), and CAT and APX activities were determined based on decomposition of H_2_O_2_ (Chance and Maehly, 1955).

### RNA Extraction and Illumina Sequencing

Total RNA was isolated from leaf tissues using the RNAprep Pure Plant Kit (TIANGEN Biotech, Beijing, China) according to the manufacturer’s instructions and then treated with DNase I (NEB) to remove DNA. Quantification was performed using a Qubit RNA Assay Kit and a Qubit 2.0 Fluorimeter (Life Technologies, CA, United States). To ensure the integrity of the RNA, we used an RNA Nano 6000 Assay Kit of the Agilent Bioanalyzer 2100 system (Agilent Technologies, CA, United States) to library construction and sequencing.

Twenty-four libraries were constructed using the NEBNext Ultra RNA Library Prep Kit for Illumina (NEB, United States). Sequences were conducted by the Biomarker Technology Company (Beijing, China) on the Illumina Hiseq™ 4,000 platform (Illumina, San Diego, CA, United States). The raw reads were first processed through in-house Perl scripts, and clean reads were obtained by moving adapter ploy-N and low-quality reads. The clean reads were mapped to the ribosome RNA (rRNA) database using the short reads alignment tool Bowtie2 (version 2.2.8), the rRNA mapped reads were removed, and the remaining clean reads were mapped to the *M. sativa* L. reference genome^1^ using HISAT2.2.4. For further analysis, all transcripts were annotated in the Nr, Nt, Swiss-Prot, Pfam, KOG/COG, KEGG, and GO databases.

### Differential Expression Analysis

Differentially expressed genes (DEGs) were analyzed using pairwise samples (G01_G02_G03 vs. G11_G12_G13, G01_G02_G03 vs. G21_G22_G23, G01_G02_G03 vs. G31_G32_G33, W01_W02_W03 vs. W11_W12_W13, W01_W02_W03 vs. W21_W22_W23, and W01_W02_W03 vs. W31_W32_W33) by DEseq R package (1.18.0). The expression levels were adjusted with FDR < 0.05, and |log_2_ (fold change) | ≥1 as a significantly differential expression.

### Trend Analysis of DEGs in “Gannong NO.3” and “WL326GZ”

To under the DEG trends, the DEGs of each variety at each sampling time were clustered by similar expression patterns. Briefly, the expression of each DEG was input, normalized, and clustered using Short Time-series Expression Miner software (STEM) with |log_2_ (fold change)| ≥1, *p* ≤ 0.05, and profile number ≤20. Then, the DEGs of each profile were annotated using the Gene Ontology (GO) and KEGG databases analysis using a *Q* ≤ 0.05.

### Weighted Gene Co-expression Network Analysis

The co-expression network was performed using the weighted gene co-expression network analysis (WGCNA) in the R software package (Langfelder and Horvath, 2008). A total of 23,977 genes with FPKM values above nine were selected for the WGCNA unsigned analysis. The adjacency matrix was constructed with a soft threshold power of six by pairwise Pearson’s correlation coefficients between pairs of genes. Then, a dissimilarity measure based on the topological overlap matrix (TOM) was used to cluster genes into the network modules on their co-expression. The module eigengene E (as the first principal component of a given module) was constructed and used as a weight to calculate the correlation of the module. The networks were visualized using Cytoscape v.3.8.0 to analyze the key modules and hub genes.

## Results

### Phenotypic Changes and Responses of Antioxidant Enzymes Following Exposure to Freezing Stress

**Figure 1:**
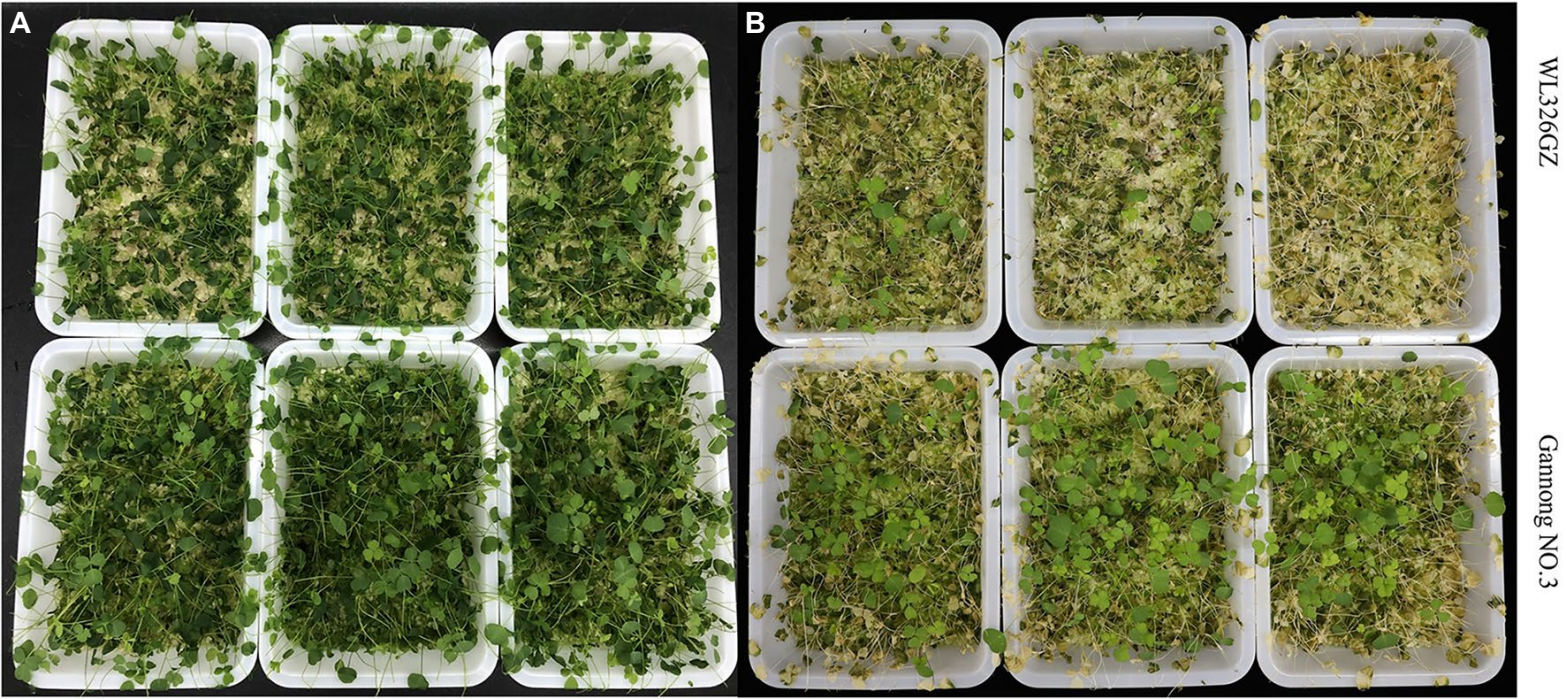
Phenotypes of two alfalfa cultivars after exposure to −10°C. **(A)** The phenotype of “WL326GZ” and “Gannong NO.3” exposure to −10°C for 2 h. **(B)** The phenotype of the **(A)** growth in the normal temperature for 2 days.

To distinguish the two different freezing tolerance types of alfalfa, we analyzed the antioxidant enzymes and phenotypic changes in the leaves following exposure to freezing stress for 0, 0.5, 1, and 2 h. The alfalfas in “Gannong NO.3” were survived more than in the “WL326GZ” under the direct exposure to −10°C temperatures for 2 h (Figure 1A). To further exhibit the phenotypic changes, we recovered the alfalfas at a normal growth temperature for 2 days (Figure 1B). Interestingly, 3.33% of the “WL326GZ” were survived, whereas 9.33% in “Gannong NO.3” (Figure 1B), indicating a higher tolerance in “Gannong NO.3,” comparing to “WL326GZ.”

**Figure 2:**
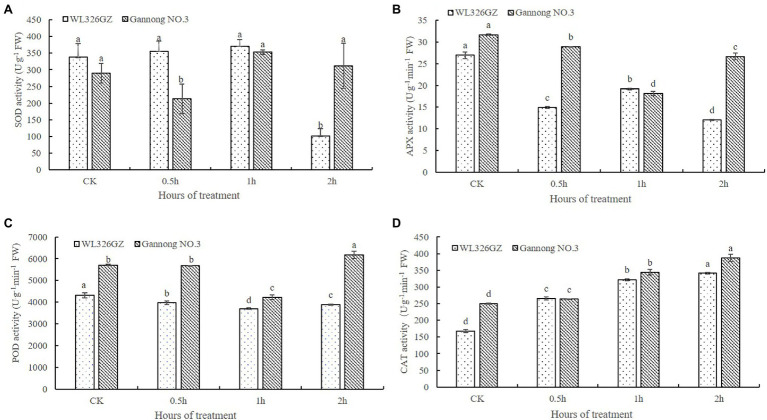
Effect of freezing stress on the activities of SOD **(A)**, APX **(B)**, POD **(C)**, and CAT **(D)** in leaves of two types of alfalfa. The data represents mean ± SD (*n* = 3), and the different letters above the bar represents the values were significant difference in “WL326GZ” or “Gannong NO.3” with different freezing treatment (*p* < 0.05, Duncan’s test).

In order to detect the effects on the freezing stress, we analyze the SOD, POD, APX, and CAT activities of the alfalfas. As shown in Figure 2A, there was no significant change of the SOD activity in “WL326GZ” under freezing stress from 0 to 1 h exposure and dramatically reduced 70.11% at 2 h, whereas the SOD activity of “Gannong NO.3” was decreased at 0.5 h and then increased 7.51% at 2 h exposure to freezing stress. The change in POD activity was similar between the “WL326GZ” and “Gannong NO.3,” both of which decreased as the freezing stress time increased, and naturally increased at 2 h. Importantly, “Gannong NO.3” has a greater extent of POD activity to “WL326GZ” at 2 h of freezing stress (Figure 2B). The variation in APX activity was similar to POD, but that of “WL326GZ” increased at 1 h, and that of “Gannong NO.3” increased at 2 h. The APX activity of “Gannong NO.3” was therefore higher than in “WL326GZ” at 2 h (Figure 2C). The CAT activity of the two types of alfalfa cultivars increased consistently with the duration of the freezing stress time, and the CAT activity of “Gannong NO.3” was higher than that of “WL326GZ” (Figure 2D). Taken together, the antioxidant activities of the sensitive cultivar “WL326GZ” occurred earlier in contrast to the tolerant type of “Gannong NO.3.”

### Bioinformatic Analysis of Differential Expressed Genes in “Gannong NO.3” and “WL326GZ”

To investigate which resistance-related genes can be beneficial to improve the freezing stress, we used RNA-seq approach to profile the leaves of “Gannong NO.3” and “WL326GZ” at 0, 0.5, 1, and 2 h exposure to −10°C. Our preliminary RNA-seq screen generated 155,133,490,050 raw data points. The raw data from each sample consisted of more than 59,000,000 data points, and all raw data were deposited in NCBI with the accession number PRJNA769225. Importantly, we cleared the raw data with GC < 43%, Q20 > 97%, and Q30 > 94%, and finally obtained 153,834,608,062 data points (Supplementary Table S1). To remove ribosome RNA (rRNA), we used Bowtie 2 to map the cleared data points to the (rRNA) database and got the clean reads ranged from 36,671,116 to 45,294,426 (Supplementary Table S2). We then mapped the clean reads to the alfalfa reference genome (Chen et al., 2020) and finally identified 121,366 genes from more than 89% of the mapped reads (see Supplementary Tables S3, S4).

**Figure 3:**
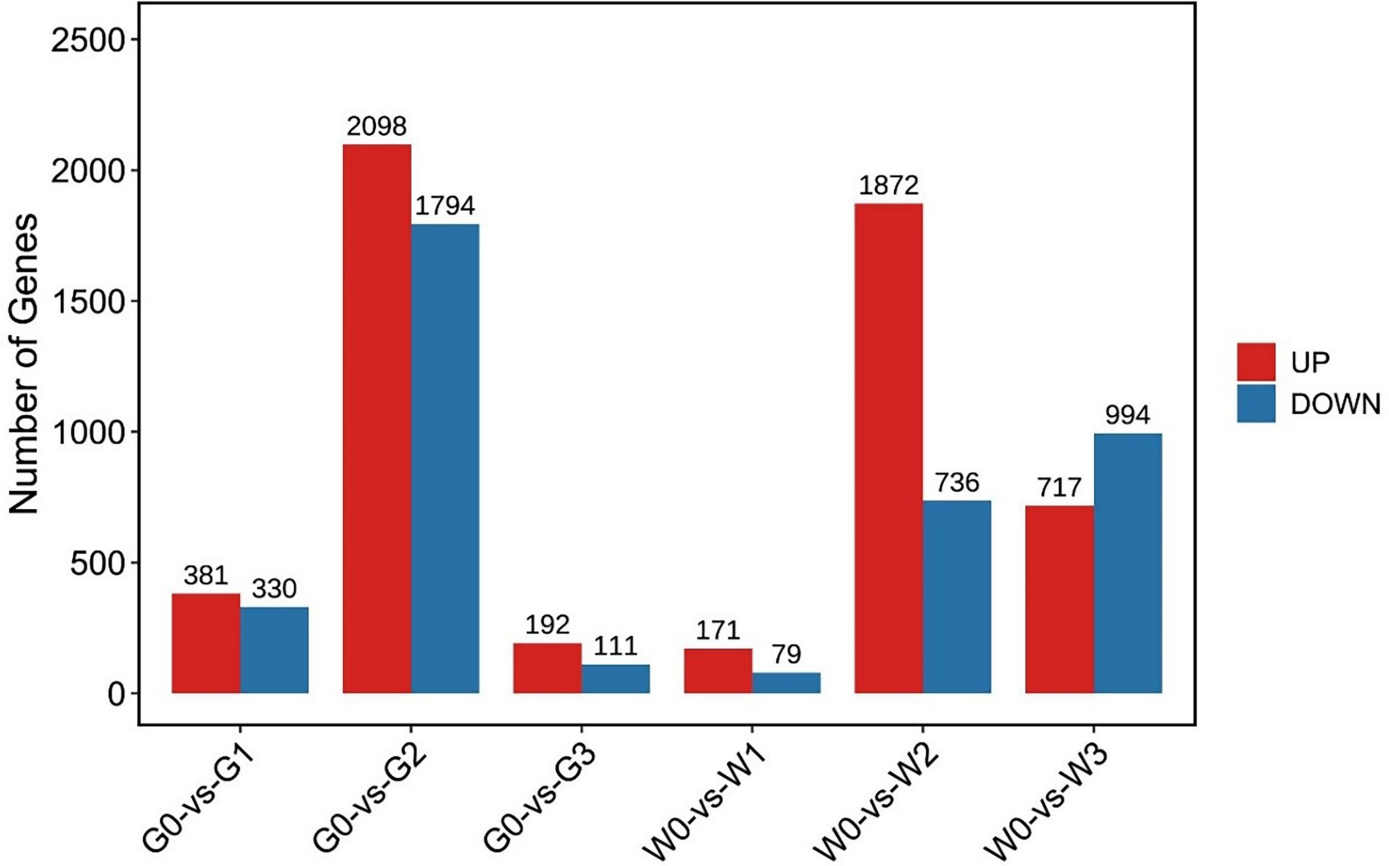
Number of differentially expressed genes, either upregulated and downregulated, in alfalfa leaves following exposure to freezing stress. G0, G1, G2, and G3 represent the samples of “Gannong NO.3” exposed to freezing stress for 0, 0.5, 1, and 2 h, respectively. W0, W1, W2, and W3 represent the samples of “WL326GZ” exposed to freezing stress for 0, 0.5, 1, and 2 h, respectively.

We then constructed a comparative analysis between freezing stress treated and control leaves of “Gannong NO.3” or “WL326GZ.” There were 7,245 DEGs that showed significant upregulation/downregulation, among that, the G0 vs. G2 and W0 vs. W2 displayed 3,892 DEGs and 2,608 DEGs, respectively (Figure 3).

### The Trend Analysis and GO and KEGG Functional Annotation of DEGs

**Figure 4:**
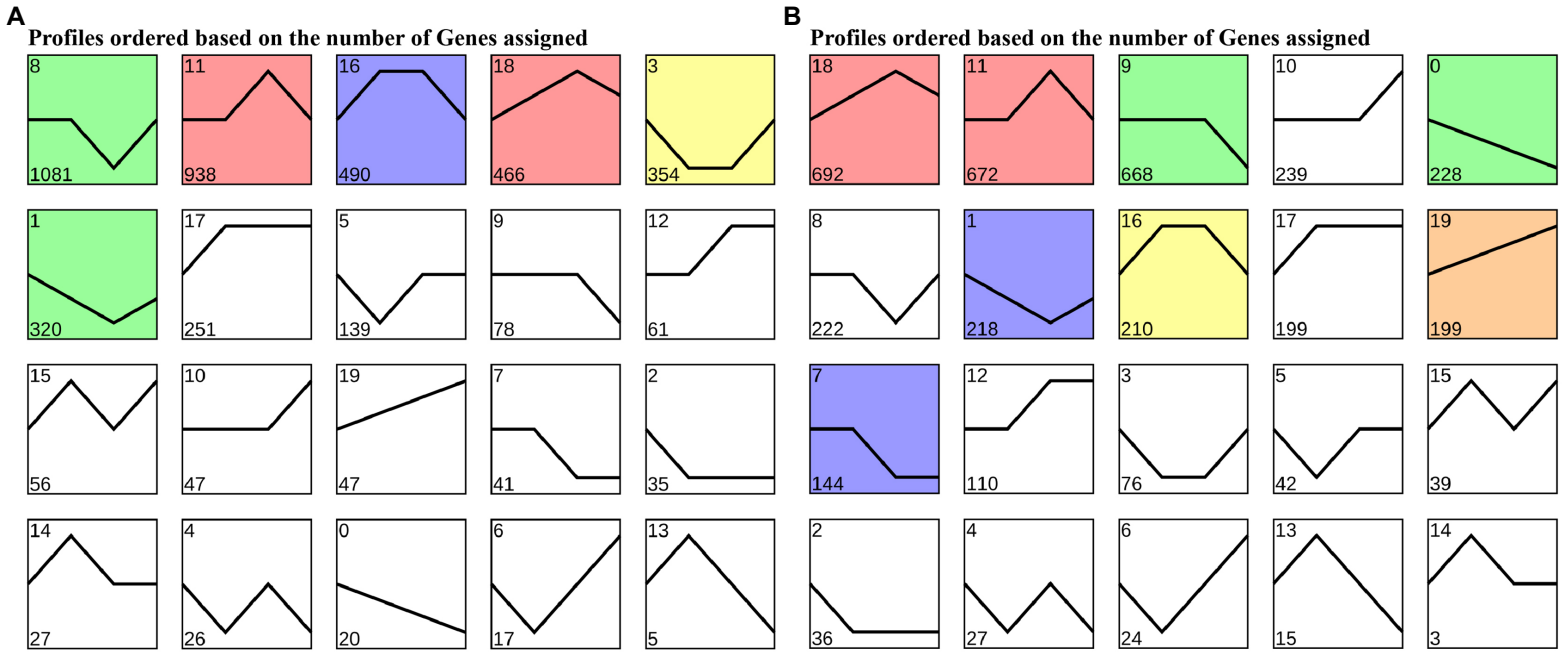
Trends in expression levels of differentially expressed genes in leaves of “Gannong NO.3” **(A)** and “WL326GZ” **(B)** in response to freezing stress.

To cluster the same trend expression genes, 20 profiles were generated using the trend analysis of “Gannong NO.3” and the top 6 profiles of the gene numbers were, 8, 11, 16, 18, 3, and 1 (Figure 4A). We then utilized the GO and KEGG pathway to identify the biological functions of the profiles. The top 20 GO enrichment (Supplementary Table S5) of the profile 8 showed 571 (81.34%) DEGs enriched in the GO:0044699 (single-organism process) and 535 (78.68%) DEGs enriched in the GO:0003824 (catalytic activity). Interestingly, profile 11 showed the most DEGs (400, 61.73%) enriched in the GO:0050896 (response to stimulus), followed by GO:0042221 (response to chemical: 270, 41.67%). As in profile 16, most DEGs (400, 61.73%) were enriched in GO:0005737 (cytoplasm), followed by GO:0044444 (cytoplasmic part). Moreover, the KEGG pathway (Supplementary Figure S1) analyses were performed for most DEGs enriched in the ko01100 (metabolic pathways) and ko01110 (biosynthesis of secondary metabolites) of profiles 8, 11, and 16.

Similar to “Gannong NO.3.,” there were 20 profiles were produced for “WL326GZ” by trend analyzed and the top 6 profiles of the DEGs were, 18, 11, 9, 10, 0, and 8 (Figure 4B). The results of GO enrichment show that 291 (67.67%) DEGs were enriched in GO:0050896 (response to stimulus), 219 (50.93%) DEGs were enriched in GO:0065007 (biological regulation) of profile 18, 264 (66.17%) DEGs were enriched in GO: 0050896 (response to stimulus), 204 (51.13%) DEGs were enriched in GO:0065007 (biological regulation) of profile 11, 315 (77.40%) DEGs were enriched in GO:0071704 (organic substance metabolic process), and 307 (75.43%) DEGs were enriched in GO:0044237 (cellular metabolic process) of profile 9 (Supplementary Table S6). The KEGG pathway (Supplementary Figure S2) analysis revealed that the results of profiles 11 and 9 coincided with those of “Gannong NO.3”; most DEGs in profile 18 were enriched in ko01110 (biosynthesis of secondary metabolites) and ko04626 (plant-pathogen interaction).

### Construction of Weight Co-expression Network and Identification of Key Modules at Different Times

**Figure 5:**
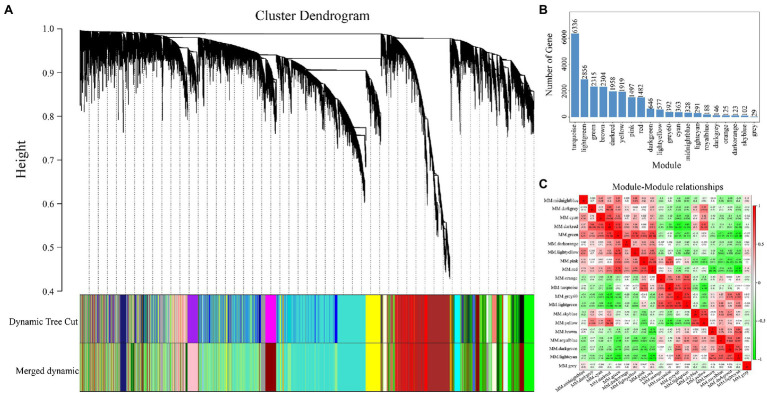
Details of the modules constructed *via* weighted gene co-expression network analysis (WGCNA). **(A)** The cluster dendrogram of the 23,977 genes. **(B)** The number of genes in each module which construct by **(A)**. **(C)** The heat map of correlation among all modules.

To construct the gene co-expression network of RNA-seq, we obtained 23,977 genes from the clean data for WGCNA. As shown in Figure 5A, 20 modules were generated and each module was independently by analyzed with eigengene adjacency heat map (Figure 5C). Moreover, the most genes from WGCNA were enriched in the turquoise module (6,336 genes, Figure 5B), followed by the light green module (2,856 genes, Figure 5B).

**Figure 6:**
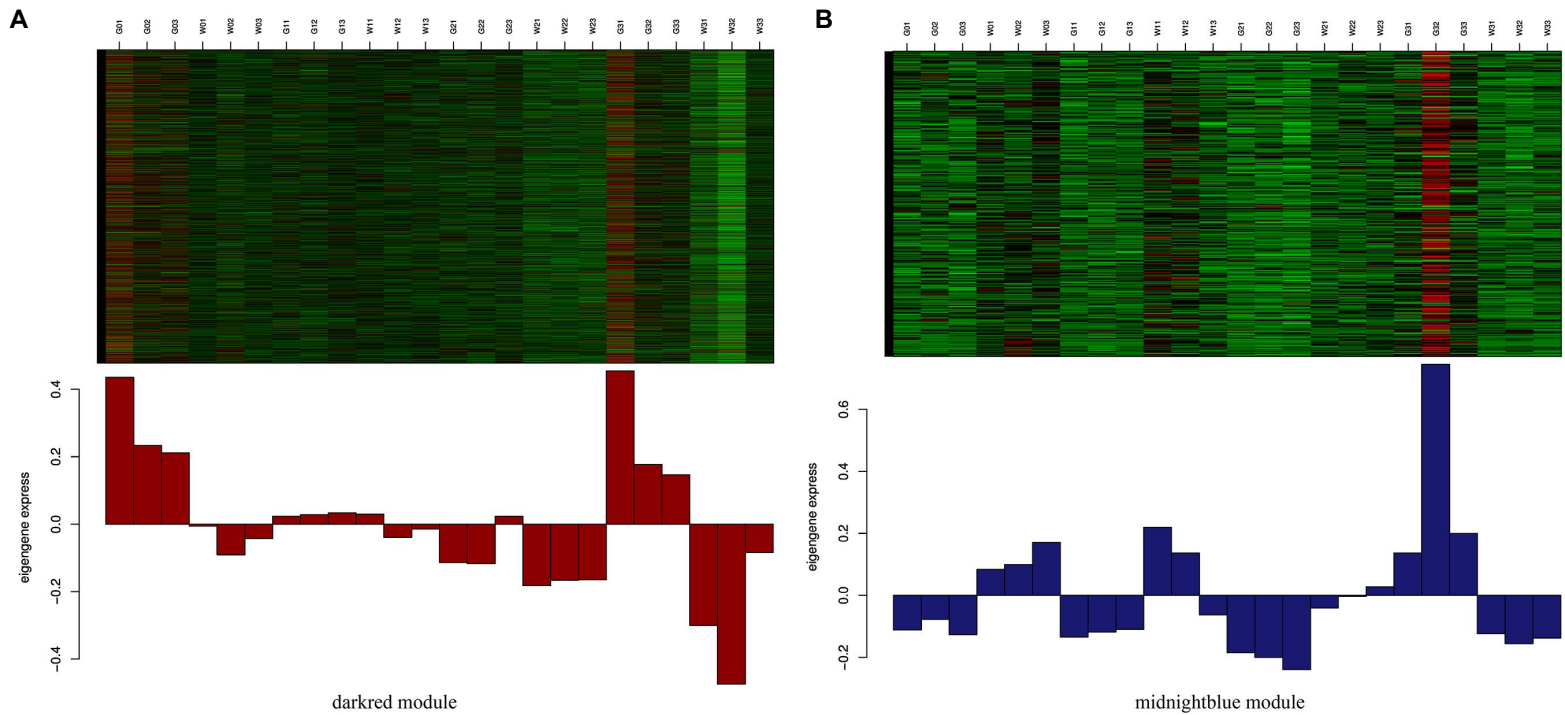
Expression levels of genes from darkred module **(A)** and midnightblue module **(B)**. Those between the darkred and midnightblue modules are most significantly correlated with “Gannong NO.3” and “WL326GZ” following exposure to freezing stress.

To identify the different reactions between “Gannong NO.3” and “WL326GZ” to the abrupt freezing stress, we obtained two modules with opposite expression patterns in those modules at different times (Figure 6). Obviously, the dark red and midnight blue modules showed opposite expressions after exposure to freezing stress.

### Function Enrichment Analysis of the Two Key Modules

To analyze the biological function of the genes from dark red and midnight blue modules, we utilized GO enrichment and KEGG pathways to classify the genes. The results of top 20 GO showed that the genes in the dark red (Supplementary Table S7) module were enriched in GO:0044699 (single-organism process), GO:0044763 (single-organism cellular process), GO:0044710 (single-organism metabolic process), GO:0050896 (response to stimulus), and GO:0044444 (cytoplasmic part), which coincide with Supplementary Table S5. The genes of the midnight blue module were enriched in GO:0005737 (cytoplasm), GO:0044444 (cytoplasmic part), GO:0044710 (single-organism metabolic process), GO:0044281 (small molecule metabolic process), and GO:0009536 (plastid). Importantly, the KEGG pathway (Supplementary Figure S3) analysis showed that most genes in the two modules were enriched in ko01100 and ko01110, which is consistent with the results of KEGG pathway in “Gannong NO.3” (Supplementary Figure S1).

### Validation of the Hub Genes of DEGs in Two Modules of the Key Pathway in KEGG

**Figure 7:**
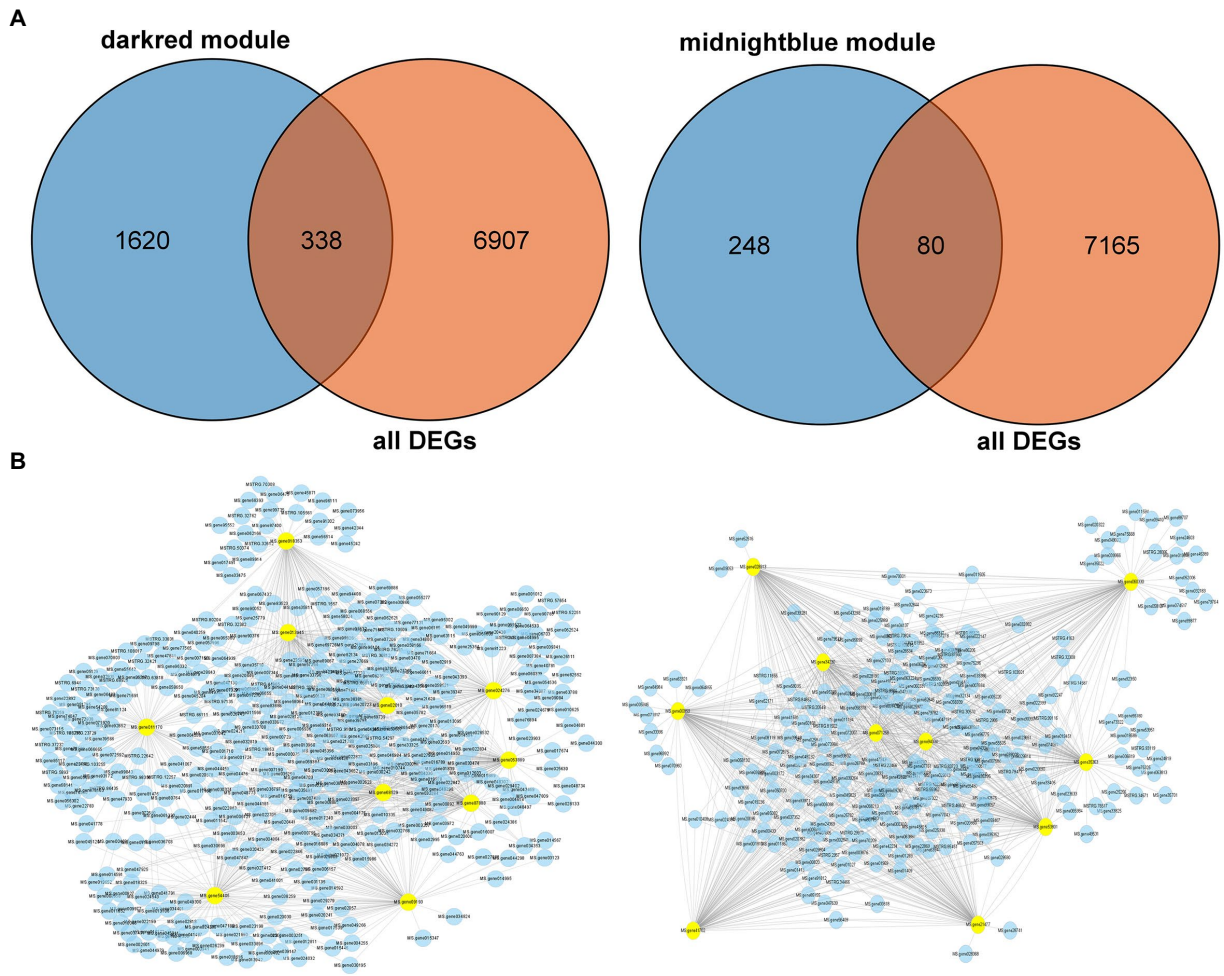
Identification the hub genes of differentially expressed genes (DEGs) in the two key modules. **(A)** Venn diagram between the two modules and all DEGs. **(B)** Correlation networks of hub genes corresponding to the darkred and midnightblue modules, respectively. One hundred genes of edge weight in the top 10 hub genes are visualized using Cytoscape.

To validate the hub genes as DEGs, we set the cutoff as |log_2_ (fold change)| ≥1 and *Q* < 0.05 to screen the DEGs. There were 7,245 DEGs identified in Figure 7A, and we overlapped the DEGs and the genes in the two modules. A total of 338 genes were observed between all DEGs and the dark red module, furthermore we chose the top 10 genes with relatively high connectivity values in the module as candidate hub genes (all.kWithin value, Figure 7B; Table 1). The results of the hub genes demonstrated that MS.gene011170 was one of the most important genes in the dark red module with highest connectivity value; its gene name is *ABCC8* [ATP-binding cassette (ABC) transporter C family member 8], which belongs to the ABC transporter C subfamily. MS.gene68529 (ABC transporter C family member 3 isoform X2, *ABCC3*), as another important candidate hub gene, also belongs to the ABC transporter C family, which indicates that the ABC transporter C subfamily may play an important role in freezing stress. However, there were only 80 DEGs identified between the genes of entire set and the midnight blue module (Figure 7A). And the top 10 genes as candidate hub genes which based on its top 10 of all.kWithin values showed that MS.gene00859 (BGLU47, beta-glucosidase 46) was one of the most critical genes activated during freezing stress (Figure 7B; Table 1).

**Table 1:** The details of 10 hub genes from the two key modules.

Gene name	All.kWithin	Description
*Darkred module*		
MS.gene011170	148.03	ABC transporter C family member 8
MS.gene013945	143.24	4-alpha-glucanotransferase DPE2
MS.gene54405	142.70	Clathrin interactor EPSIN 3
MS.gene87888	138.31	Very-long-chain aldehyde decarbonylase CER3-like isoform X1
MS.gene053889	136.06	Peptide/nitrate transporter
MS.gene09193	130.04	Lipoxygenase 6, chloroplastic
MS.gene024276	123.06	Light-regulated protein 1, chloroplastic
MS.gene018353	120.55	Pathogenesis-related protein PR-1
MS.gene68529	119.15	ABC transporter C family member 3 isoform X2
MS.gene02010	118.06	Ferric reduction oxidase 7, chloroplastic
*Midnightblue module*		
MS.gene00859	24.24	Beta-glucosidase 46
MS.gene94034	21.13	D-pinitol dehydrogenase
MS.gene41702	19.69	NAC transcription factor 47
MS.gene53801	18.02	B2 protein
MS.gene071258	17.61	Serine protease SPPA, chloroplastic
MS.gene35363	16.41	Histone deacetylase HDT1
MS.gene21477	14.10	Cytochrome P450 family ent-kaurenoic acid oxidase
MS.gene028913	13.53	E3 ubiquitin-protein ligase RMA1H1
MS.gene060330	13.09	Pyrophosphate-energized vacuolar membrane proton pump
MS.gene34230	12.80	Probable aminotransferase TAT2 isoform X1

### Freezing Stress Induces Numerous DEGs in the ABC Family

**Figure 8:**
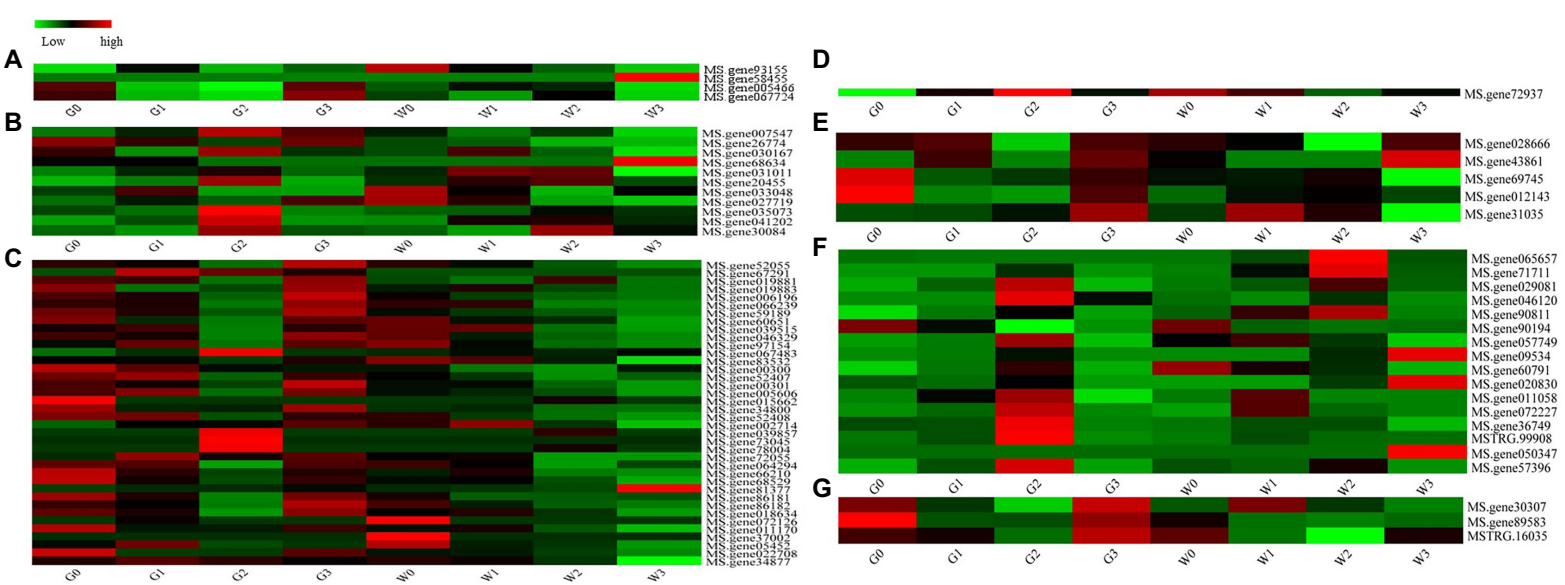
Heat maps of the ATP-binding cassette (ABC) family genes in the alfalfas from RNA-seq. **(A)** ABC Subfamily A, **(B)** ABC Subfamily B, **(C)** ABC Subfamily C, **(D)** ABC Subfamily E, **(E)** ABC Subfamily F, **(F)** ABC Subfamily G, and **(G)** ABC Subfamily I.

The ABC transporter family is one of the largest transporter families in living organisms because of its important physiological functions (Nguyen et al., 2014). In our study, totally of 80 DEGs belonged to the ABC gene family, and they were grouped into seven subfamilies (Figure 8). A heat map of 80 DEGs indicates (Figure 8) that four genes (5.00%) belong to ABC subfamily A, 11 genes (13.75%) belong to ABC subfamily B, 39 genes (48.75%) belong to ABC subfamily C, one gene (1.25%) belongs to ABC subfamily E, five genes (6.25%) belong to ABC subfamily F, 17 genes (21.25%) belong to ABC subfamily G, and three genes (3.75%) belong to ABC subfamily I. Obviously, ABC subfamily C plays a critical role in freezing stress response.

Furthermore, we also compared the 39 genes of ABC subfamily C to the database of the WGCNA, which indicated that there were 17 genes included in the database (Table 2). And analyzed the relatively high connectivity values of 17 genes, the results indicated that the highest values of genes were MS.gene011170 and MS.gene68529 that could be acted as hub genes of ABC subfamily C in all modules (All.kTotal value), which coincides with the results of Table 1.

**Table 2:** The results of overlapped genes between ABCC subfamily genes and differentially expressed genes (DEGs).

Gene ID	Module	All.kTotal
MS.gene011170	Darkred	719.0367653
MS.gene68529	Darkred	616.0700359
MS.gene006196	Green	585.8495929
MS.gene59189	Green	557.947855
MS.gene00301	Green	541.9193836
MS.gene066239	Lightyellow	541.5092946
MS.gene52055	Lightyellow	487.9046613
MS.gene52408	Green	464.8650029
MS.gene34800	Darkred	452.5614335
MS.gene046329	Lightyellow	390.2598191
MS.gene81377	Turquoise	357.9430749
MS.gene005606	Dark red	305.3770089
MS.gene039515	Lightyellow	294.0799577
MS.gene97154	Lightyellow	269.936822
MS.gene72055	Darkred	144.6371911
MS.gene66210	Darkred	91.06072232
MS.gene002714	Lightgreen	75.9891247

### Expression Profiles of the Putative Complex Network of Signal Transduction

**Figure 9:**
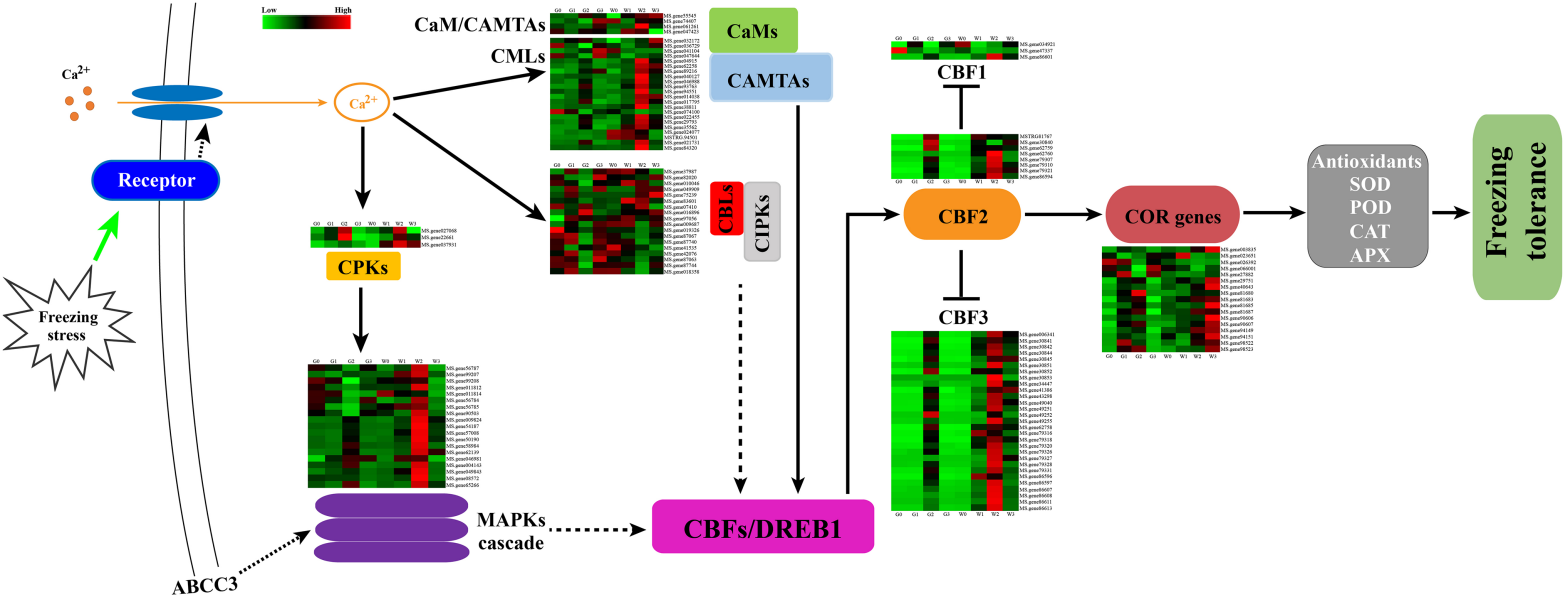
Hypothetical schematic diagram for the mechanism of tolerance to abrupt freezing stress in alfalfa. Freezing stress induced calcium signal transduction through CaMs, CMLs, CBLs, and CPKs. CPKs directly responded to the signal transduction and active MAPKs were involved in the downstream CBFs/DREB1 expression. CaMs and CBLs interacted with CAMTAs and CIPKs, respectively, and regulated the genes of CBFs/DREB1. CBF1, CBF2, and CBF3 trigged an expression of COR genes, which resulted in an increase in the activities of SOD, POD, CAT, and APX. We hypothesize that the hub gene ABCC3 was involved in the MAPK signal pathway and regulated the related genes of CBFs expression. This may have triggered the COR genes and led to an increase in antioxidant enzymes that counteracted abrupt freezing stress in the alfalfa leaves. The dashed arrows indicate that the mechanism is unclear, and CBF2 reduced the expression of CBF1 and CBF3.

Freezing stress could cause osmotic stress in plant cells and rapidly trigger Ca^2+^ signal transduction, which could induce several Ca^2+^ signal transduction-related genes expressed (Gong et al., 2020). In this study, CaMs/CMLs, CAMATs CBLs, CIPKs, CPKs, and MAPKs were induced during the freezing stress (Figure 9). Furthermore, most of the DEGs of the CaMs/CMLs-CAMATs increased over the freezing time and then decreased. And freezing stress induced the expression of most genes in G2 and W2. In contrast, only three (MS.gene036729, MS.gene047644, and MS.gene074100) decreased from G0 to G2 and W0 to W1 as well as increased to G3 and W3. Interestingly, the similar change of CBLs-CIPK for “Gannong NO.3” showed a kick point at G2, but that of “WL326GZ” was at W1 Whereas, there only were three DEGs belonged to CPKs. And all DEGs consistently increased at G2 and W2 and then decreased. Furthermore, CPKs could activated the MAPK cascade expressed to regulate the cold responses in plants. In our study, the changes of the MS.genes 56787, 99208, 011812, 56784, 56785, and 90503 genes belongs to MAPK casacade which were decreased from G0 to G2 and increased at G3; whereas in “WL326GZ,” the genes were increased from W0 to W2 and then decreased at W3. And the variation of the other DEGs was similar between “Gannong NO.3” and “WL326GZ.” Importantly, the DEGs of “WL326GZ” showed a greater expression than those of “Gannong NO.3.” And all the DEGs of CaMs-CAMTAs, CBLs-CIPKs, and the MAPK cascade were induced *via* CBFs/DREB1s (C-repeat binding factors/dehydrate responsive element binding factors) to confer freezing stress.

### Expression of CBFs/DREB1s Related Genes Induced by Abrupt Freezing

The CBF/DREB1 network of genes is important in mitigating freezing stress (Cheng et al., 2007), especially *CBF1/DREB1B*, *CBF2/DREB1C*, and *CBF3/DREB1A* (Ullah et al., 2017). In our study, the expression of *CBF1/DREB1B*, *CBF2/DREB1C*, and *CBF3/DREB1A* genes in alfalfa leaves was higher after freezing stress (Figure 9) and was associated with the expression of COR genes. Importantly, the most genes of CBFs/DREB1 of “WL326GZ” were induced earlier than in “Gannong NO.3” leaves.

## Discussion

### Physiological Responses of Leaves in Two Different Types of Alfalfa to Freezing Stress

Freezing stress induces ROS (e.g., superoxide radicals, hydrogen peroxide, and hydrogen radicals), which damages the cell membrane and increases lipid peroxidation in plants. Plants activate antioxidant defense systems, including SOD, CAT, POD, and APX, to alleviate the damage caused by ROS (Liang et al., 2008). It is demonstrated that the functions of SOD, POD, and APX convert the superoxide radical into hydrogen peroxide, and CAT eliminated hydrogen peroxide (Wang et al., 2009). Recently, several reports have demonstrated that ROS levels are negatively related to stress resistance, and the greater the stress resistance of plants, the stronger the activities of the antioxidant system (Peng et al., 2021). Importantly, our results (Figure 2) are in accordance with this conclusion, as the activities of antioxidant in “Gannong NO.3” leaves were higher than those in “WL326GZ” leaves. Similarly, antioxidant enzyme levels of other freezing tolerant plants have consistently been higher than in their freezing-sensitive counterparts for many species, including maize (Hodges et al., 1997), rice (Huang and Guo, 2005), and chickpeas (Kaur et al., 2009). Furthermore, alfalfa cultivar showed similar trends to those of our study (Wang et al., 2009). Therefore, our conclusions suggest that alfalfa acquires freezing tolerance by increasing the activities of antioxidant enzymes in the ROS scavenging system.

### Differentially Expressed Genes in Response to Freezing Stress

To explore the changes in abrupt freezing stress-related gene expression in alfalfa cultivars, we transcribed RNA from alfalfa leaves which exposure to −10°C for 0, 0.5, 1, and 2 h. In this study, we obtained 121,366 genes by RNA-seq based on the alfalfa reference genome. Our transcription assembly was more efficient than that of other alfalfa studies (Song et al., 2016; Shu et al., 2017; Xu et al., 2019). Furthermore, 7,245 DEGs were identified from the leaves of “Gannong NO.3” and “WL326GZ” exposure to freezing stress. To compare the differences in response to freezing stress in the two alfalfa cultivars, we analyzed the trends of DEGs in “Gannong NO.3” and “WL326GZ.” These results suggest that metabolic pathways and biosynthesis of secondary metabolites may play a critical role in counteracting freezing stress. Moreover, the GO enrichment results showed DEGs have been enriched in response to stimulus and stress in biological processes and in the binding and catalytic activities during molecular functions, which were been included in the transcriptomics for several other alfalfa cultivars of freezing stress (Shu et al., 2017; Xu et al., 2019).

### Role of ABC Transporter Family Genes in Abrupt Freezing Tolerance

The ABC transporter proteins are one of the largest protein families and found in yeast, mammals, and plants (Raichaudhuri, 2016). And several studies have revealed that ABC proteins play a key role in plants involved in diverse processes, including abiotic stress. In plants, eight ABC transporter subfamilies have been classified, subfamilies ABAA-G and ABCI (Wanke and Üner Kolukisaoglu, 2010). In our study, we identified 80 DEGs belonging to the ABC family, which were grouped into seven subfamilies exclude ABCD subfamily (Figure 8). The evidence showed that ABCD subfamily transporters are involved in the development of related peroxisomes in *Caenorhabditis elegans*, which have been previously found in insects (Tian et al., 2017); thus, there were no ABCD subfamily genes induced in our study. Interestingly, there were 39 genes (48.75%) belonging to the ABCC subfamily, which suggests that this subfamily may play a critical role in freezing tolerance. Importantly, as Raichaudhuri (2016) reported that the ABCC subfamily is one of the most important subfamilies of ABC transporters based on it can transport many types of glutathione conjugates in vacuolar membranes. And ABCCs have been identified as glutathione-S (GS) conjugate pumps, which can transport endogenous substances and chlorophyll catabolites as well as regulate the ion channel (Wanke and Üner Kolukisaoglu, 2010). Thus, the ABCC subfamily occupies an important position in response to freezing stress.

To analyze the hub genes as DEGs in alfalfa leaves in response to freezing stress, we obtained two hub genes belonging to the ABCC subfamily (Table 1), *ABCC8* and *ABCC3*. Multiple *ABCC8* genes have been observed in plants, such as *AtABCC8* (Sánchez-Fernández et al., 2001), *OsABCC8* (Jasinski et al., 2003), *FvABCC8* (Shi et al., 2020), and *TaABCC8* (Bhati et al., 2015). However, only a few studies have reported the function of *ABCC8* genes in plants. Recently, *SmABCC8* was found to be involved in the transportation of substances in leaves (Yan et al., 2021). Moreover, *OsABCC8* has been identified as a homolog of *AtABCC4* in rice, which is associated with the response of guard cell plasma membrane ion channels to abiotic stress (Saha et al., 2015). Therefore, we hypothesize that *ABCC8* has a similar function in alfalfa.

In addition, recent studies have shown that *ZmMRP3* (*ZmABCC3*) participates in the biosynthesis of anthocyanin (Rea, 2007), and overexpression of *AtABCC3* improves tolerance to bleomycin (Li et al., 2021) and SA. Moreover, *ABCC3* is also associated with the MAPK signaling pathway (Guo et al., 2015; Jin et al., 2020), which can be resistant to biotic (Guo et al., 2019), and thus is important for adaptation to environmental changes (Brunetti et al., 2015; Saha et al., 2015; Su et al., 2021). Therefore, the alfalfa *ABCC3* may help regulate downstream genes *via* the MAPK signal pathway to contribute to freezing tolerance (Figure 9).

### Ca^2+^ Signal Transduction Components Trigger Downstream CBF/DREB1 Gene Expression for Resistance to Freezing Stress

Calcium ions (Ca^2+^) act as the second messenger involved in a wide range of biological processes in eukaryotic cells and plays a key role in counteracting cold stress. Interestingly, in alfalfa, Orvar et al. (2000) reported that cold stress-induced rigidification at microdomains on the plasma membrane results in a rearrangement of the cytoskeleton; this leads to stretch-sensitive Ca^2+^ channels and elevates the concentration of Ca^2+^in the cytosol, which stimulates expression of cold-induced genes to resist stress. Similar Ca^2+^ signals were induced in this study. Ca^2+^ signals are relayed or respond to Ca^2+^ sensor proteins (Ogunrinde et al., 2017), including CaMs, CMLs, and CBLs belonging to sensor relays, and CPKs/CDPKs that acted as sensor responders in this study. CaMs/CMLs and CBLs do not have any catalytic activity; their functions need to be combined with biomolecular interactions. For example, CaMs combine with calmodulin-binding transcriptional activators (CAMTAs) and CBL-interacting protein kinases (CIPKs) to relay Ca^2+^ transients for phosphorylation (Viswanathan and Zhu, 2002; Marcec et al., 2019), and CMLs can interact with many types of proteins (Perochon et al., 2011).

We found that the CaMs/CMLs-CAMTAs DEGs increased with prolonged freezing stress and then decreased. Thus, CaMs-CAMTAs may positively regulate freezing tolerance. Our results are consistent with those of other studies (Kim et al., 2013; Yuan et al., 2018; Yang et al., 2020). CAMTAs positively regulate CBFs and CORs (Kim et al., 2013), thereby increasing the activity of the ROS scavenging system, including POD, CAT, and APX, to defend against freezing stress (Peng et al., 2021). However, the gene expression of CBLs-CIPKs is reportedly more complex than that of CaMs-CAMTAs (Zhang et al., 2020). One CBL can function with one CIPK or multiple CIPKs, and the final function of CIPKs depends on interactions with CBLs, which can participate in multiple biological processes (Tang et al., 2020). Thus, they are expressed in different tissues under various stresses, including cold stress (Yuan et al., 2018). Therefore, several genes of CBLs-CIPKs were expressed in this study. The responses of CPKs/CDPKs proteins to the Ca^2+^ sensor play an important role in the Ca^2+^ signals involved in ion channels, metabolic enzymes, and other processes (Yip Delormel and Boudsocq, 2019). In alfalfa, CPKs/CDPKs prevent cold-induced gene expression, preventing plants from adapting to environmental changes (Monroy et al., 1993); this is consistent with our results. The expression of CPKs in cold-sensitive “WL326GZ” increased more rapidly than in cold-resistant “Gannong NO.3,” ultimately resulting in a much higher expression in the former. In addition, CDPKs can induce the MAPK pathways in response to cold stress (Sangwan et al., 2002; Lv et al., 2018). MAPK cascades are key in alleviating adverse environmental conditions. For example, many MAPKs are involved in the cascade process (Zhang and Klessig, 2001); MMK4 in alfalfa is induced under cold stress (Jonak et al., 1996); and MPK3 and MPK6 reduce CBF gene expression to negatively regulate cold resistance (Zhao et al., 2017). Similar to these reports, we found that several MAPKs were induced by abrupt freezing stress. And Sangwan et al. (2002) demonstrated that cold shock-induced MAPKs in alfalfa to resist freezing tolerance, which is consistent with our results. Overall, the components of Ca^2+^ signal transduction triggered downstream CBFs/DREB1 gene expression in alfalfa to ensure resistance to freezing stress (Figure 9).

CBF/DREB1 belongs to the APETALA2/ethylene-responsive factor (AP2/ERF) transcription factor family (Yang et al., 2009), which is also critical in mitigating cold stress based on initially induced cold response transcript genes in a transcriptional cascade (Kidokoro et al., 2017); moreover, it plays a key role in facilitating COR and CBF gene expression (Yang et al., 2009). In Arabidopsis, *CBF1* (*DREB1B*), *CBF2* (*DREB1C*), and *CBF3* (*DREB1A*) were rapidly induced under cold stress, and overexpression of *CBF1, CBF2,* and *CBF3* increased freezing tolerance after exposure to freezing shock stress (Park et al., 2020). Moreover, alfalfa CBFs have been identified as key regulators of freezing tolerance (Anower et al., 2016). In this study, the expression levels of CBF2 and CBF3 genes initially increased at 1 h of freezing stress and then decreased (Figure 9). This dynamic has been identified in Arabidopsis (Daniel et al., 2003). The expression levels of CBF2 and CBF3 in “WL326GZ” were higher and occurred earlier than those in “Gannong NO.3,” possibly because the former is sensitive to abrupt freezing stress. This is accordance with our observed trends for the antioxidant enzyme activities. However, our results are contrary to the cold acclimation mechanism of alfalfa (Chen et al., 2015). This indicates that the abrupt freezing stress response mechanism is different from that of cold acclimation.

In summary, we compared the leaves of the freeze-tolerant genotype “Gannong NO.3” and the freeze-sensitive genotype “WL326GZ” after exposure to abrupt freezing stress and evaluated their physiology and transcriptome. Our results suggest that the ABCC subfamily plays an important role in tolerance to abrupt freezing stress in alfalfa. Moreover, we demonstrated that *ABCC8* and *ABCC3* are the key genes in counteracting freezing stress, and further studies should explore the exact functions of these genes. A simple working module is illustrated in Figure 9. The expression levels of the Ca^2+^ signal transduction and CBF/DREB1 related genes demonstrate their critical role in counteracting freezing stress. Additionally, they may be associated with the higher antioxidant enzyme activities (SOD, POD, CAT, and APX activities) in “Gannong NO.3” than in “WL326GZ” (Figure 9). This could explain why “WL326GZ” is more sensitive to abrupt changes in temperature than “Gannong NO.3.” Moreover, high antioxidant activities early in the stress exposure were associated with high expression levels of genes related to freezing tolerance, even though the duration of the high antioxidant activities was short. Our results provide new insights into the mechanisms of abrupt freezing tolerance in alfalfa.

## Data Availability Statement

The datasets presented in this study can be found in online repositories. The names of the repository/repositories and accession number(s) can be found in the article/Supplementary Material.

## Author Contributions

JM and SS conceived and designed the research. XW, WK, and FW conducted the experiment. XW and JM analyzed the data and wrote the manuscript. WK, FW, and SS finalized the manuscript. All authors contributed to the article and approved the submitted version.

## Funding

This work was supported by the Fostering Foundation for the Excellent Ph.D. Dissertation of Gansu Agricultural University (YB2019001), Major Special Science and Technology Projects of Gansu Province: Germplasm Innovation and Breeding of Alfalfa and Oat (19ZD2NA002-3), and Discipline Construction Fund Project of Gansu Agricultural University (GAU-XKJS-2018-002).

## Conflict of Interest

The authors declare that the research was conducted in the absence of any commercial or financial relationships that could be construed as a potential conflict of interest.

## Publisher’s Note

All claims expressed in this article are solely those of the authors and do not necessarily represent those of their affiliated organizations, or those of the publisher, the editors and the reviewers. Any product that may be evaluated in this article, or claim that may be made by its manufacturer, is not guaranteed or endorsed by the publisher.

## Footnotes

^1^ https://figshare.com/projects/whole_genome_sequencing_and_assembly_of_Medicago_sativa/66380

## Supplementary Material

Supplementary Figure S1 KEGG pathway of the top 3 profiles in the “Gannong NO.3” leaf samples.

Supplementary Figure S2 KEGG pathway of the top 3 profiles in the “WL326GZ” leaf samples.

Supplementary Figure S3 Bar plot of the top 20 KEGG pathways in the two key modules.

## References

[ref1] Ahmed N. U. Jung H. J. Park J. I. Cho Y. G. Hur Y. Nou I. S. (2015). Identification and expression analysis of cold and freezing stress responsive genes of Brassica oleracea. Gene 554, 215–223. doi: 10.1016/j.gene.2014.10.050, PMID: 25445291

[ref3] Anower M. R. Fennell A. Boe A. Mott I. W. Peel M. D. Wu Y. (2016). Physiological and molecular characterisation of lucerne (*Medicago sativa* L.) germplasm with improved seedling freezing tolerance. Crop Pasture Sci. 67:655. doi: 10.1071/cp15204

[ref4] Bhati K. K. Sharma S. Aggarwal S. Kaur M. Shukla V. Kaur J.. (2015). Genome-wide identification and expression characterization of ABCC-MRP transporters in hexaploid wheat. Front. Plant Sci. 6:488. doi: 10.3389/fpls.2015.00488, PMID: 26191068 PMC4486771

[ref5] Brunetti P. Zanella L. De Paolis A. Di Litta D. Cecchetti V. Falasca G.. (2015). Cadmium-inducible expression of the ABC-type transporter AtABCC3 increases phytochelatin-mediated cadmium tolerance in *Arabidopsis*. J. Exp. Bot. 66, 3815–3829. doi: 10.1093/jxb/erv185, PMID: 25900618 PMC4473984

[ref6] Castonguay Y. Rocher S. Bertrand A. Michaud J. B. (2020). Identification of transcripts associated with the acquisition of superior freezing tolerance in recurrently-selected populations of alfalfa. Euphytica 216:27. doi: 10.1007/s10681-020-2559-2

[ref7] Chance B. Maehly A. C. (1955). Assay of catalases and peroxidases. Methods Enzymol. 2, 764–775. doi: 10.1016/S0076-6879(55)02300-8 13193536

[ref8] Chao Y. Yuan J. Guo T. Xu L. Mu Z. Han L. (2019). Analysis of transcripts and splice isoforms in *Medicago sativa* L. by single-molecule long-read sequencing. Plant Mol. Biol. 99, 219–235. doi: 10.1007/s11103-018-0813-y, PMID: 30600412

[ref9] Chen J. Han G. Q. Shang C. Li J. K. Zhang H. L. Liu F. Q.. (2015). Proteomic analyses reveal differences in cold acclimation mechanisms in freezing-tolerant and freezing-sensitive cultivars of alfalfa. Front. Plant Sci. 6:105. doi: 10.3389/fpls.2015.00105, PMID: 25774161 PMC4343008

[ref10] Chen J. Xue B. Xia X. Yin W. (2013). A novel calcium-dependent protein kinase gene from Populus euphratica, confers both drought and cold stress tolerance. Biochem. Biophys. Res. Commun. 441, 630–636. doi: 10.1016/j.bbrc.2013.10.103, PMID: 24177011

[ref11] Chen H. Zeng Y. Yang Y. Huang L. Tang B. Zhang H.. (2020). Allele-aware chromosome-level genome assembly and efficient transgene-free genome editing for the autotetraploid cultivated alfalfa. Nature. Communications 11:2494. doi: 10.1038/s41467-020-16338-x, PMID: 32427850 PMC7237683

[ref12] Cheng C. Yun K. Y. Ressom H. W. Mohanty B. Bajic V. B. Jia Y.. (2007). An early response regulatory cluster induced by low temperature and hydrogen peroxide in seedlings of chilling-tolerant japonica rice. BMC Genomics 8:175. doi: 10.1186/1471-2164-8-175, PMID: 17577400 PMC1925099

[ref13] Cheong Y. H. Kim K. N. Pandey G. K. Gupta R. Grant J. J. Luan S. (2003). CBL1, a calcium sensor that differentially regulates salt, drought, and cold responses in *Arabidopsis*. Plant Cell 15, 1833–1845. doi: 10.1105/tpc.012393, PMID: 12897256 PMC167173

[ref14] Cho K. M. Nguyen H. T. K. Kim S. Y. Shin J. S. Cho D. H. Hong S. B.. (2016). CML10, a variant of calmodulin, modulates ascorbic acid synthesis. New Phytol. 209, 664–678. doi: 10.1111/nph.13612, PMID: 26315131

[ref15] Daniel G. Z. Jonathan T. Vogel C. D. Thomashow M. F. (2003). Cold induction of *Arabidopsis* CBF genes involves multiple ICE (inducer of CBF expression) promoter elements and a cold-regulatory circuit that is desensitized by low temperature. Plant Physiol. 133, 910–918. doi: 10.1104/pp.103.027169 14500791 PMC219064

[ref16] Delk N. A. Johnson K. A. Chowdhury N. I. Braam J. (2005). *CML24*, regulated in expression by diverse stimuli, encodes a potential Ca^2+^ sensor that functions in responses to abscisic acid, daylength, and ion stress. Plant Physiol. 139, 240–253. doi: 10.1104/pp.105.062612, PMID: 16113225 PMC1203374

[ref17] Dubrovina A. S. Kiselev K. V. Khristenko V. S. Aleynova O. A. (2015). *VaCPK20,* a calcium-dependent protein kinase gene of wild grapevine Vitis amurensis Rupr., mediates cold and drought stress tolerance. J. Plant Physiol. 185, 1–12. doi: 10.1016/j.jplph.2015.05.020, PMID: 26264965

[ref18] Eremina M. Rozhon W. Poppenberger B. (2016). Hormonal control of cold stress responses in plants. Cell. Mol. Life Sci. 73, 797–810. doi: 10.1007/s00018-015-2089-6, PMID: 26598281 PMC11108489

[ref19] Giannopolitis C. N. Ries S. K. (1977). Superoxide dismutases I. Occurrence in higher plants. Plant Physiol. 59, 309–314. doi: 10.1104/pp.59.2.309, PMID: 16659839 PMC542387

[ref20] Gong Z. Z. Xiong L. M. Shi H. Z. Yang S. H. Herrera-Estrella L. R. Xu G.. (2020). Plant abiotic stress response and nutrient use efficiency. Sci. China Life Sci. 63, 635–674. doi: 10.1007/s11427-020-1683-x 32246404

[ref21] Guo Z. Kang S. Chen D. Wu Q. Wang S. Xie W.. (2015). MAPK signaling pathway alters expression of Midgut ALP and ABCC genes and causes resistance to bacillus thuringiensis Cry1Ac toxin in diamondback moth. PLoS Genet. 11:e1005124. doi: 10.1371/journal.pgen.1005124, PMID: 25875245 PMC4395465

[ref22] Guo Z. Sun D. Kang S. Zhou J. Gong L. Qin J.. (2019). CRISPR/Cas9-mediated knockout of both the PxABCC2 and PxABCC3 genes confers high-level resistance to *Bacillus thuringiensis* Cry1Ac toxin in the diamondback moth, *Plutella xylostella* (L.). Insect Biochem. Mol. Biol. 107, 31–38. doi: 10.1016/j.ibmb.2019.01.009, PMID: 30710623

[ref23] Hodges D. M. Andrews C. J. Johnson D. A. Hamilton R. I. (1997). Antioxidant enzyme responses to chilling stress in differentially sensitive inbred maize lines. J. Exp. Bot. 48, 1105–1113. doi: 10.1093/jxb/48.5.1105

[ref24] Huang M. Guo Z. (2005). Responses of antioxidative system to chilling stress in two rice cultivars differing in sensitivity. Biol. Plant. 49, 81–84. doi: 10.1007/s00000-005-1084-3 17098438

[ref25] Jasinski M. Ducos E. Martinoia E. Boutry M. (2003). The ATP-binding cassette transporters: structure, function, and gene family comparison between rice and Arabidopsis. Plant Physiol. 131, 1169–1177. doi: 10.1104/pp.102.014720, PMID: 12644668 PMC1540298

[ref26] Jin M. Yang Y. Shan Y. Chakrabarty S. Cheng Y. Soberón M.. (2020). Two ABC transporters are differentially involved in the toxicity of two bacillus thuringiensis Cry1 toxins to the invasive crop-pest *Spodoptera frugiperda* (J. E. Smith). Pest Manag. Sci. 77, 1492–1501. doi: 10.1002/ps.6170, PMID: 33145907

[ref27] Jonak C. Kiegerl S. Ligterink W. Barker P. J. Huskisson N. S. Hirt H. (1996). Stress signaling in plants: a mitogen-activated protein kinase pathway is activated by cold and drought. Plant Biol. 93, 111274–111279. 10.1073/pnas.93.20.11274 PMC38320 8855346

[ref28] Kanchupati P. Wang Y. Anower M. R. Boe A. Wu Y. (2017). The CBF-like gene family in alfalfa: expression analyses and identification of potential functional homologs of *Arabidopsis* CBF3. Crop Sci. 57, 2051–2063. doi: 10.2135/cropsci2016.09.0777

[ref29] Karimzadeh S. H. Nezami A. Nabati J. Oskoueian E. Ahmadi-Lahijani M. J. (2021). The physiological, biochemical, and molecular modifications of chickpea (*Cicer arietinum* L.) seedlings under freezing stress. J. Plant Growth Regul. 1–16. doi: 10.1007/s00344-021-10369-433649694

[ref30] Kaur S. Gupta A. K. Kaur N. Sandhu J. S. Gupta S. K. (2009). Antioxidative enzymes and sucrose synthase contribute to cold stress tolerance in chickpea. J. Agron. Crop Sci. 195, 393–397. doi: 10.1111/j.1439-037X.2009.00383.x

[ref31] Kidokoro S. Yoneda K. Takasaki H. Takahashi F. Shinozaki K. Yamaguchi-Shinozaki K. (2017). Different cold-signaling pathways function in the responses to rapid and gradual decreases in temperature. Plant Cell 29, 760–774. doi: 10.1105/tpc.16.00669, PMID: 28351986 PMC5435423

[ref32] Kim Y. Park S. Gilmour S. J. Thomashow M. F. (2013). Roles of CAMTA transcription factors and salicylic acid in configuring the low-temperature transcriptome and freezing tolerance of *Arabidopsis*. Plant J. 75, 364–376. doi: 10.1111/tpj.12205, PMID: 23581962

[ref33] Kolukisaoglu Ü. Weinl S. Blazevic D. Batistic O. Kudla J. (2004). Calcium sensors and their interacting protein kinases: genomics of the arabidopsis and rice CBL-CIPK signaling networks. Plant Physiol. 134, 43–58. doi: 10.1104/pp.103.033068, PMID: 14730064 PMC316286

[ref34] Komatsu S. Yang G. Khan M. Onodera H. Toki S. Yamaguchi M. (2007). Over expression of calcium-dependent protein kinase 13 and calreticulin interacting protein 1 confers cold tolerance on rice plants. Mol. Gen. Genomics. 277, 713–723. doi: 10.1007/s00438-007-0220-6, PMID: 17318583

[ref35] Langfelder P. Horvath S. (2008). WGCNA: an R package for weighted correlation network analysis. BMC Bioinformatics 9:559. doi: 10.1186/1471-2105-9-559, PMID: 19114008 PMC2631488

[ref36] Lee J. Lim Y. P. Han C. T. Nou I. S. Hur Y. (2013). Genome-wide expression profiles of contrasting inbred lines of Chinese cabbage, Chiifu and Kenshin, under temperature stress. Genes Genomics 35, 273–288. doi: 10.1007/s13258-013-0088-2

[ref37] Li D. Song S. Xia X. Yin W. (2012). Two CBL genes from Populus euphratica confer multiple stress tolerance in transgenic triploid white poplar. Plant Cell Tissue Organ Cult. 109, 477–489. doi: 10.1007/s11240-011-0112-7

[ref38] Li T. Xu S. Wu C. Yan S. Wang L. (2021). Loss of an ABC transporter in Arabidopsis thaliana confers hypersensitivity to the anti-cancer drug bleomycin. DNA Repair 106:103174. doi: 10.1016/j.dnarep.2021.103174, PMID: 34256304

[ref39] Li H. Ye K. Shi Y. Cheng J. Zhang X. Yang S. (2017). BZR1 positively regulates freezing tolerance via CBF-dependent and CBF-independent pathways in *Arabidopsis*. Mol. Plant 10, 545–559. doi: 10.1016/j.molp.2017.01.004, PMID: 28089951

[ref40] Liang Y. Zhu J. Li Z. Chu G. Ding Y. Zhang J.. (2008). Role of silicon in enhancing resistance to freezing stress in two contrasting winter wheat cultivars. Environ. Exp. Bot. 64, 286–294. doi: 10.1016/j.envexpbot.2008.06.005

[ref41] Liu Y. Wu C. Hu X. Gao H. Wang Y. Luo H.. (2020). Transcriptome profiling reveals the crucial biological pathways involved in cold response in Moso bamboo (*Phyllostachys edulis*). Tree Physiol. 40, 538–556. doi: 10.1093/treephys/tpz133, PMID: 31860727

[ref42] Lv X. Li H. Chen X. Xiang X. Guo Z. Yu J.. (2018). The role of calcium-dependent protein kinase in hydrogen peroxide, nitric oxide and ABA-dependent cold acclimation. J. Exp. Bot. 69, 4127–4139. doi: 10.1093/jxb/ery212, PMID: 29868714 PMC6054180

[ref43] Ma Q. Zhou Q. Chen C. Cui Q. Zhao Y. Wang K.. (2019). Isolation and expression analysis of *CsCML* genes in response to abiotic stresses in the tea plant (*Camellia sinensis*). Sci. Rep. 9:8211. doi: 10.1038/s41598-019-44681-7, PMID: 31160625 PMC6547691

[ref44] Mall T. K. Dweikat I. Sato S. J. Neresian N. Xu K. Ge Z.. (2011). Expression of the rice CDPK-7 in sorghum: molecular and phenotypic analyses. Plant Mol. Biol. 75, 467–479. doi: 10.1007/s11103-011-9741-9, PMID: 21318369

[ref45] Marcec M. J. Gilroy S. Poovaiah B. W. Tanaka K. (2019). Mutual interplay of Ca^2+^ and ROS signaling in plant immune response. Plant Sci. 283, 343–354. doi: 10.1016/j.plantsci.2019.03.004, PMID: 31128705

[ref46] Monroy A. F. Dhindsa R. S. (1995). Low-temperature signal transduction: induction of cold acclimation-specific genes of alfalfa by calcium at 25 degrees C. Plant Cell 7, 321–331. doi: 10.2307/386985, PMID: 7734966 PMC160785

[ref47] Monroy A. F. Sarhan F. Dhindsa R. S. (1993). Cold induced changes in freezing tolerance, protein phosphorylation, and gene expression: evidence for a role of calcium. Plant Physiol. 102, 1227–1235. doi: 10.1104/pp.102.4.1227, PMID: 12231898 PMC158909

[ref001] Nah G. Lee M. Kim D. S. Rayburn A. L. Voigt T. Lee D. K.. (2016). Transcriptome analysis of Spartina pectinata in response to freezing stress. PLoS One 11:e0152294. doi: 10.1371/journal.pone.0152294 27032112 PMC4816275

[ref48] Nguyen V. N. T. Moon S. Jung K. H. (2014). Genome-wide expression analysis of rice ABC transporter family across spatio-temporal samples and in response to abiotic stresses. J. Plant Physiol. 171, 1276–1288. doi: 10.1016/j.jplph.2014.05.006, PMID: 25014263

[ref49] Ogunrinde A. Munro K. Davidson A. Ubaid M. Snedden W. A. (2017). *Arabidopsis* calmodulin-like proteins, CML15 and CML16 possess biochemical properties distinct from calmodulin and show non-overlapping tissue expression patterns. Front. Plant Sci. 8:2175. doi: 10.3389/fpls.2017.02175, PMID: 29312414 PMC5743801

[ref50] Orvar B. L. Sangwan V. Omann F. Dhindsa R. S. (2000). Early steps in cold sensing by plant cells: the role of actin cytoskeleton and membrane fluidity. Plant J. 23, 785–794. doi: 10.1046/j.1365-313x.2000.00845.x, PMID: 10998189

[ref51] Park S. Shi A. Mou B. (2020). Genome-wide identification and expression analysis of the CBF/DREB1 gene family in lettuce. Sci. Rep. 10:5733. doi: 10.1038/s41598-020-62458-1, PMID: 32235838 PMC7109083

[ref52] Peng T. You X. S. Guo L. Zhong B. L. Mi L. F. Chen J. M.. (2021). Transcriptome analysis of Chongyi wild mandarin, a wild species more cold-tolerant than *Poncirus trifoliata*, reveals key pathways in response to cold. Environ. Exp. Bot. 184:104371. doi: 10.1016/j.envexpbot.2020.104371

[ref53] Perochon A. Aldon D. Galaud J. P. Ranty B. (2011). Calmodulin and calmodulin-like proteins in plant calcium signaling. Biochimie 93, 2048–2053. doi: 10.1016/j.biochi.2011.07.012 21798306

[ref54] Raichaudhuri A. (2016). Arabidopsis thaliana MRP1 (*AtABCC1*) nucleotide binding domain contributes to arsenic stress tolerance with serine triad phosphorylation. Plant Physiol. Biochem. 108, 109–120. doi: 10.1016/j.plaphy.2016.07.005, PMID: 27428365

[ref55] Ray S. Agarwal P. Arora R. Kapoor S. Taygi A. K. (2007). Expression analysis of calcium-dependent protein kinase gene family during reproductive development and abiotic stress conditions in rice (*Oryza sativa* L. spp. indica). Mol. Gen. Genet. 278, 493–505. doi: 10.1007/s00438-007-0267-4, PMID: 17636330

[ref56] Rea P. A. (2007). Plant ATP-binding cassette transporters. Annu. Rev. Plant Biol. 58, 347–375. doi: 10.1146/annurev.arplant.57.032905.105406 17263663

[ref57] Saha J. Sengupta A. Gupta K. Gupta B. (2015). Molecular phylogenetic study and expression analysis of ATP-binding cassette transporter gene family in *Oryza sativa* in response to salt stress. Comput. Biol. Chem. 54, 18–32. doi: 10.1016/j.compbiolchem.2014.11.0, PMID: 25531538

[ref58] Sánchez-Fernández R. Davies T. G. E. Coleman J. O. D. Rea P. A. (2001). The *Arabidopsis thaliana* ABC protein superfamily, a complete inventory. J. Biol. Chem. 276, 30231–30244. doi: 10.1074/jbc.m103104200, PMID: 11346655

[ref59] Sangwan V. Orvar B. L. Beyerly J. Hirt H. Dhindsa R. S. (2002). Opposite changes in membrane fluidity mimic cold and heat stress activation of distinct plant MAP kinase pathways. Plant J. 31, 629–638. doi: 10.1046/j.1365-313x.2002.01384.x, PMID: 12207652

[ref60] Shi M. Wang S. Zhang Y. Wang S. Zhao J. Feng H.. (2020). Genome-wide characterization and expression analysis of ATP-binding cassette (ABC) transporters in strawberry reveal the role of *FvABCC11* in cadmium tolerance. Sci. Hortic. 271:109464. doi: 10.1016/j.scienta.2020.109464

[ref61] Shu Y. Li W. Zhao J. Zhang S. Xu H. Liu Y.. (2017). Transcriptome sequencing analysis of alfalfa reveals CBF genes potentially playing important roles in response to freezing stress. Genet. Mol. Biol. 40, 824–833. doi: 10.1590/1678-4685-gmb-2017-0053, PMID: 29111565 PMC5738619

[ref62] Song L. Jiang L. Chen Y. Shu Y. Bai Y. Guo C. (2016). Deep-sequencing transcriptome analysis of field-grown *Medicago sativa* L. crown buds acclimated to freezing stress. Funct. Integr. Genomics 16, 495–511. doi: 10.1007/s10142-016-0500-5, PMID: 27272950

[ref63] Su L. Xie Y. He Z. Zhang J. Tang Y. Zhou X. (2021). Network response of two cherry tomato (*Lycopersicon esculentum*) cultivars to cadmium stress as revealed by transcriptome analysis. Ecotoxicol. Environ. Saf. 222:112473. doi: 10.1016/j.ecoenv.2021.112473, PMID: 34224970

[ref64] Sun Q. Huang R. Zhu H. Sun Y. Guo Z. (2021). A novel *Medicago truncatula* calmodulin-like protein (MtCML42) regulates cold tolerance and flowering time. Plant J. 108, 1069–1082. doi: 10.1111/tpj.15494, PMID: 34528312

[ref65] Sun T. Wang Y. Wang M. Li T. Zhou Y. Wang X.. (2015). Identification and comprehensive analyses of the CBL, and CIPK, gene families in wheat (*Triticum aestivum* L). Plant Biol. 15:269. doi: 10.1186/s12870-015-0657-4, PMID: 26537110 PMC4634908

[ref66] Tang R.-J. Wang C. Li K. Luan S. (2020). The CBL-CIPK calcium signaling network: unified paradigm from 20 years of discoveries. Trends Plant Sci. 25, 604–617. doi: 10.1016/j.tplants.2020.01.009, PMID: 32407699

[ref67] Tayeh N. Bahrman N. Sellier H. Bluteau A. Blassiau C. Fourment J.. (2013). A tandem array of CBF/DREB1 genes is located in a major freezing tolerance QTL region on *Medicago truncatula* chromosome 6. BMC Genomics 14:814. doi: 10.1186/1471-2164-14-814, PMID: 24261852 PMC4046650

[ref68] Tian L. Song T. He R. Zeng Y. Xie W. Wu Q.. (2017). Genome-wide analysis of ATP-binding cassette (ABC) transporters in the sweetpotato whitefly, *Bemisia tabaci*. BMC Genomics 18:330. doi: 10.1186/s12864-017-3706-6, PMID: 28446145 PMC5405539

[ref69] Townley H. E. Knight M. R. (2002). Calmodulin as a potential negative regulator of *Arabidopsis* COR gene expression. Plant Physiol. 128, 1169–1172. doi: 10.1104/pp.010814 11950965

[ref70] Tsutsui T. Kato W. Asada Y. Sako K. Sato T. Sonoda Y.. (2009). DEAR1, a transcriptional repressor of DREB protein that mediates plant defense and freezing stress responses in *Arabidopsis*. J. Plant Res. 122, 633–643. doi: 10.1007/s10265-009-0252-6, PMID: 19618250

[ref71] Ullah J. A. Fazal H. Midrarullah A. A. Khaista R. (2017). Role of CBF/DREB gene expression in abiotic stress tolerance. A review symbiosis list of abbreviations. Int. J. Hort. Agric. 2, 1–12.

[ref72] Viswanathan C. Zhu J. K. (2002). Molecular genetic analysis of cold-regulated gene transcription. Philos. Trans. R. Soc. Lond. B. Biol. Sci. 357, 877–886. doi: 10.1098/rstb.2002.1076, PMID: 12171651 PMC1693007

[ref73] Wang L. Feng X. Yao L. Ding C. Lei L. Hao X.. (2020). Characterization of CBL–CIPK signaling complexes and their involvement in cold response in tea plant. Plant Physiol. Biochem. 154, 195–203. doi: 10.1016/j.plaphy.2020.06.005, PMID: 32563043

[ref74] Wang W. B. Kim Y. H. Lee H. S. Deng X. P. Kwak S. S. (2009). Differential antioxidation activities in two alfalfa cultivars under chilling stress. Plant Biotechnol. Rep. 3, 301–307. doi: 10.1007/s11816-009-0102-y

[ref75] Wanke D. Üner Kolukisaoglu H. (2010). An update on the ABCC transporter family in plants: many genes, many proteins, but how many functions? Plant Biol. 12, 15–25. doi: 10.1111/j.1438-8677.2010.00380.x, PMID: 20712617

[ref76] Weckwerth P. Ehlert B. Romeis T. (2015). ZmCPK1, a calcium-independent kinase member of the Zea mays CDPK gene family, functions as a negative regulator in cold stress signalling. Plant Cell Environ. 38, 544–558. doi: 10.1111/pce.12414 25052912

[ref77] Xu L. Tang X. Wang B. Xin X. Guo M. (2019). Comparative transcriptome analysis of five medicago varieties reveals the genetic signals underlying freezing tolerance. Crop Pasture Sci. 70:273. doi: 10.1071/CP18165

[ref78] Yan L. Zhang J. Chen H. Luo H. (2021). Genome-wide analysis of ATP-binding cassette transporter provides insight to genes related to bioactive metabolite transportation in Salvia miltiorrhiza. BMC Genomics 22:315. doi: 10.1186/s12864-021-07623-0, PMID: 33933003 PMC8088630

[ref79] Yang T. Chaudhuri S. Yang L. Du L. Poovaiah B. W. (2009). A calcium/calmodulin-regulated member of the receptor-like kinase family confers cold tolerance in plants. J. Biol. Chem. 285, 7119–7126. doi: 10.1074/jbc.m109.035659, PMID: 20026608 PMC2844161

[ref80] Yang F. Dong F. Hu F. Liu Y. Chai J. Zhao H.. (2020). Genome-wide identification and expression analysis of the calmodulin-binding transcription activator (CAMTA) gene family in wheat (*Triticum aestivum* L.). BMC Genet. 21:105. doi: 10.1186/s12863-020-00916-5, PMID: 32928120 PMC7491182

[ref81] Yang T. B. Poovaiah B. W. (2003). Calcium/calmodulin-mediated signal network in plants. Trends Plant Sci. 8, 505–512. doi: 10.1016/j.tplants.2003.09.004, PMID: 14557048

[ref82] Yang S. S. Xu W. Tesfaye M. Lamb J. F. Jung H. J. G. VandenBosch K. A.. (2010). Transcript profiling of two alfalfa genotypes with contrasting cell wall composition in stems using a cross-species platform: optimizing analysis by masking biased probes. BMC Genomics 11:323. doi: 10.1186/1471-2164-11-323, PMID: 20497574 PMC2893600

[ref83] Yip Delormel T. Boudsocq M. (2019). Properties and functions of calcium-dependent protein kinases and their relatives in *Arabidopsis thaliana*. New Phytol. 224, 585–604. doi: 10.1111/nph.16088 31369160

[ref84] Yuan P. Yang T. Poovaiah B. W. (2018). Calcium signaling-mediated plant response to cold stress. Int. J. Mol. Sci. 19:3896. doi: 10.3390/ijms19123896, PMID: 30563125 PMC6320992

[ref85] Zeng H. Xu L. Singh A. Wang H. Du L. Poovaiah B. W. (2015). Involvement of calmodulin and calmodulin-like proteins in plant responses to abiotic stresses. Front. Plant Sci. 6:600. doi: 10.3389/fpls.2015.00600, PMID: 26322054 PMC4532166

[ref86] Zhang S. Klessig D. F. (2001). MAPK cascades in plant defense signaling. Trends Plant Sci. 6, 520–527. doi: 10.1016/s1360-1385(01)02103-3 11701380

[ref87] Zhang F. Li L. Jiao Z. Chen Y. Liu H. Chen X.. (2016). Characterization of the calcineurin B-like (CBL) gene family in maize and functional analysis of ZmCBL9 under abscisic acid and abiotic stress treatments. Plant Sci. 253, 118–129. doi: 10.1016/j.plantsci.2016.09.011, PMID: 27968980

[ref88] Zhang X. Li X. Zhao R. Zhou Y. Jiao Y. (2020). Evolutionary strategies drive a balance of the interacting gene products for the CBL and CIPK gene families. New Phytol. 226, 1506–1516. doi: 10.1111/nph.16445, PMID: 31967665

[ref89] Zhao C. Wang P. Si T. Hsu C. C. Wang L. Zayed O.. (2017). MAP kinase cascades regulate the cold response by modulating ICE1 protein stability. Dev. Cell 43, 618.e5–629.e5. doi: 10.1016/j.devcel.2017.09.024, PMID: 29056551 PMC5716877

[ref90] Zhou Y. Cheng Y. Yang Y. Li X. Supriyo B. Sun X.. (2016). Overexpression of SpCBL6, a calcineurin B-like protein of *Stipa purpurea*, enhanced cold tolerance and reduced drought tolerance in transgenic *Arabidopsis*. Mol. Biol. Rep. 43, 957–966. doi: 10.1007/s11033-016-4036-5, PMID: 27393148

[ref91] Zhou Q. Luo D. Chai X. Wu Y. Wang Y. Nan Z.. (2018). Multiple regulatory networks are activated during cold stress in *Medicago sativa* L. Int. J. Mol. Sci. 19:3169. doi: 10.3390/ijms19103169, PMID: 30326607 PMC6214131

[ref92] Zhu X. Dunand C. Snedden W. Galaud J. P. (2015). CaM and CML emergence in the green lineage. Trends Plant Sci. 20, 483–489. doi: 10.1016/j.tplants.2015.05.010, PMID: 26115779

