# Supplementary figures and images for: Comparative Transcriptome Analysis Reveals New Insight of Alfalfa (Medicago sativa L.) Cultivars in Response to Abrupt Freezing Stress
Source: Front Plant Sci. 2022 Mar 31;13:798118. doi: 10.3389/fpls.2022.798118 (PMC9010130; doi:10.3389/fpls.2022.798118)

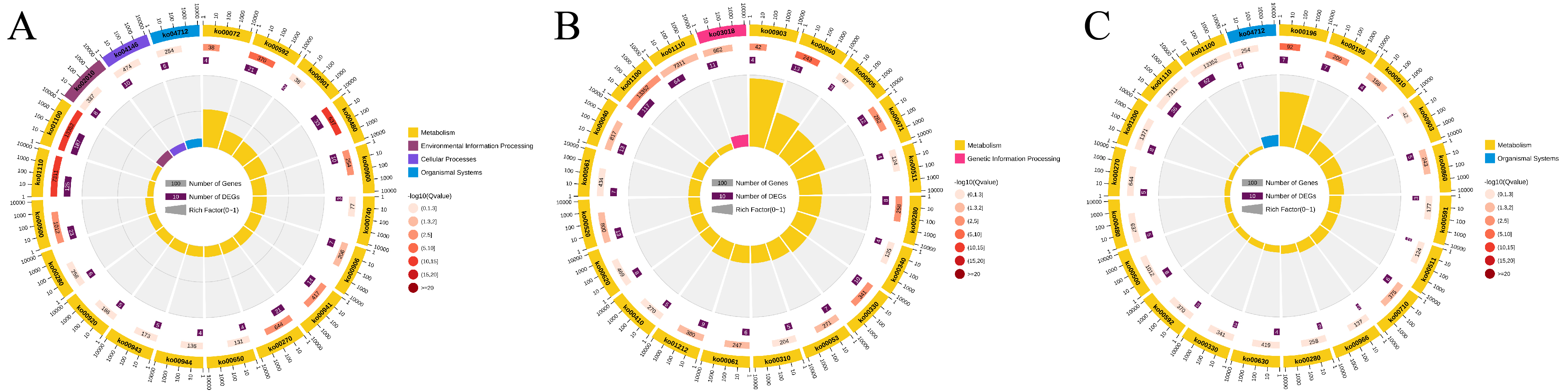

Supplement: Supplementary Figure S1 — KEGG pathway of the top 3 profiles in the “Gannong NO.3” leaf samples. [file Figure_10.JPEG]

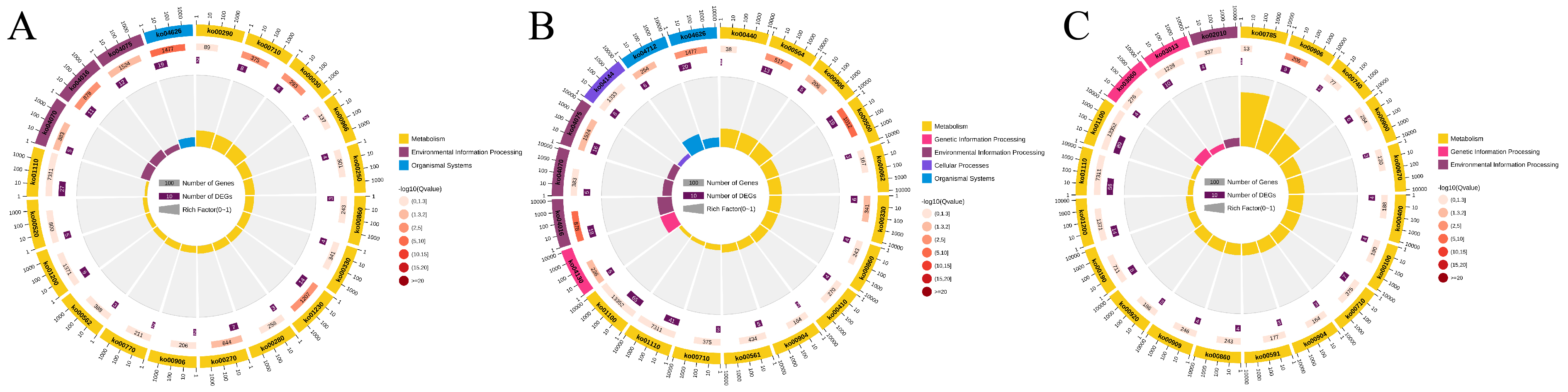

Supplement: Supplementary Figure S2 — KEGG pathway of the top 3 profiles in the “WL326GZ” leaf samples. [file Figure_11.JPEG]

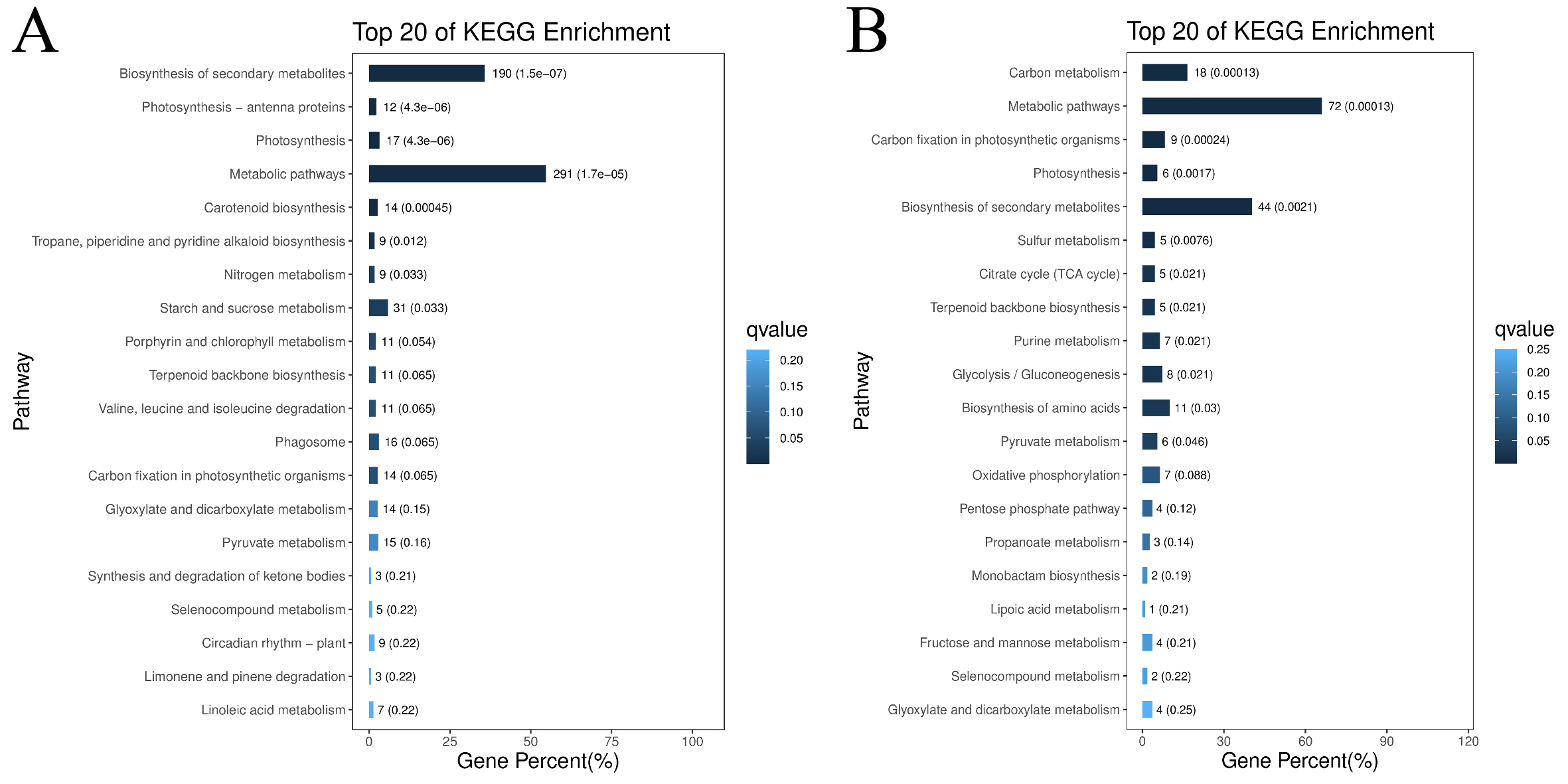

Supplement: Supplementary Figure S3 — Bar plot of the top 20 KEGG pathways in the two key modules. [file Figure_12.JPEG]
