# Supplementary material for: Comparative Transcriptome Analysis Reveals New Insight of Alfalfa (Medicago sativa L.) Cultivars in Response to Abrupt Freezing Stress
Source: Front Plant Sci. 2022 Mar 31;13:798118. doi: 10.3389/fpls.2022.798118 (PMC9010130; doi:10.3389/fpls.2022.798118)
Supplement: Supplementary file 4 [file Table_1.docx]

Supplements

Table S1 Details of the raw data from the 24 leaf samples.

| Sample | RawData (bp) | BF_Q20 (%) | BF_Q30 (%) | BF_N (%) | BF_GC (%) | CleanData (bp) | AF_Q20 (%) | AF_Q30 (%) | AF_N (%) | AF_GC (%) |
| --- | --- | --- | --- | --- | --- | --- | --- | --- | --- | --- |
| G01 | 6599578294 | 6462150953 (97.92%) | 6219508660 (94.24%) | 84582 (0.00%) | 2827223417 (42.84%) | 6537377953 | 6420312490 (98.21%) | 6183211408 (94.58%) | 83718 (0.00%) | 2794321757 (42.74%) |
| G02 | 6512342762 | 6372737110 (97.86%) | 6129990816 (94.13%) | 86881 (0.00%) | 2759106464 (42.37%) | 6456109342 | 6334733929 (98.12%) | 6097003202 (94.44%) | 86198 (0.00%) | 2729745622 (42.28%) |
| G03 | 6588119066 | 6449139158 (97.89%) | 6205472917 (94.19%) | 90111 (0.00%) | 2795599984 (42.43%) | 6533792238 | 6412455778 (98.14%) | 6173692809 (94.49%) | 89311 (0.00%) | 2767164364 (42.35%) |
| G11 | 5946762706 | 5816239428 (97.81%) | 5596083126 (94.10%) | 85511 (0.00%) | 2527210851 (42.50%) | 5891683737 | 5779294798 (98.09%) | 5564299259 (94.44%) | 84594 (0.00%) | 2498281561 (42.40%) |
| G12 | 5973729626 | 5848646464 (97.91%) | 5631580158 (94.27%) | 73595 (0.00%) | 2593704095 (43.42%) | 5916326542 | 5810457217 (98.21%) | 5598657826 (94.63%) | 72835 (0.00%) | 2563143601 (43.32%) |
| G13 | 6684555698 | 6553055597 (98.03%) | 6312486420 (94.43%) | 86381 (0.00%) | 2812354770 (42.07%) | 6635997349 | 6520070913 (98.25%) | 6283716166 (94.69%) | 85702 (0.00%) | 2787320334 (42.00%) |
| G21 | 6233106456 | 6098579920 (97.84%) | 5862681516 (94.06%) | 88721 (0.00%) | 2636218637 (42.29%) | 6181800227 | 6063844025 (98.09%) | 5832655173 (94.35%) | 88014 (0.00%) | 2609595882 (42.21%) |
| G22 | 6126082728 | 5984920025 (97.70%) | 5748900415 (93.84%) | 87446 (0.00%) | 2592709952 (42.32%) | 6067873736 | 5945882688 (97.99%) | 5715296764 (94.19%) | 86657 (0.00%) | 2562153368 (42.22%) |
| G23 | 6709304146 | 6561938647 (97.80%) | 6312743450 (94.09%) | 91045 (0.00%) | 2894765681 (43.15%) | 6636995244 | 6512913968 (98.13%) | 6270095402 (94.47%) | 90105 (0.00%) | 2855736492 (43.03%) |
| G31 | 6691592880 | 6550474189 (97.89%) | 6304922770 (94.22%) | 88954 (0.00%) | 2836508182 (42.39%) | 6627112143 | 6506641394 (98.18%) | 6266679419 (94.56%) | 88011 (0.00%) | 2802466407 (42.29%) |
| G32 | 6612648118 | 6469840699 (97.84%) | 6222962808 (94.11%) | 86614 (0.00%) | 2803470147 (42.40%) | 6546003203 | 6424868425 (98.15%) | 6183804698 (94.47%) | 85611 (0.00%) | 2768513702 (42.29%) |
| G33 | 6500274070 | 6359248712 (97.83%) | 6117371815 (94.11%) | 83845 (0.00%) | 2743610240 (42.21%) | 6437561618 | 6316663644 (98.12%) | 6080123801 (94.45%) | 83031 (0.00%) | 2710724196 (42.11%) |
| W01 | 6547269740 | 6416956046 (98.01%) | 6179506997 (94.38%) | 86848 (0.00%) | 2759705250 (42.15%) | 6496851227 | 6382518256 (98.24%) | 6149504309 (94.65%) | 86126 (0.00%) | 2733707190 (42.08%) |
| W02 | 6749357840 | 6609107315 (97.92%) | 6358833385 (94.21%) | 89739 (0.00%) | 2843111892 (42.12%) | 6698346903 | 6574412941 (98.15%) | 6328675487 (94.48%) | 89020 (0.00%) | 2817009244 (42.06%) |
| W03 | 5907162610 | 5782042481 (97.88%) | 5560665419 (94.13%) | 85947 (0.00%) | 2494932856 (42.24%) | 5865295237 | 5753276744 (98.09%) | 5535607638 (94.38%) | 85391 (0.00%) | 2473234180 (42.17%) |
| W11 | 6622888008 | 6487380476 (97.95%) | 6241745902 (94.25%) | 89817 (0.00%) | 2777743761 (41.94%) | 6577410851 | 6456117164 (98.16%) | 6214501925 (94.48%) | 89227 (0.00%) | 2754454557 (41.88%) |
| W12 | 6845913798 | 6710985164 (98.03%) | 6462687613 (94.40%) | 90516 (0.00%) | 2890103645 (42.22%) | 6793214095 | 6674830489 (98.26%) | 6431053456 (94.67%) | 89795 (0.00%) | 2863044055 (42.15%) |
| W13 | 6682105378 | 6544631310 (97.94%) | 6302331881 (94.32%) | 88837 (0.00%) | 2848775681 (42.63%) | 6620848179 | 6502909439 (98.22%) | 6265890565 (94.64%) | 87919 (0.00%) | 2816517911 (42.54%) |
| W21 | 6329431764 | 6207039938 (98.07%) | 5989169350 (94.62%) | 90930 (0.00%) | 2697438387 (42.62%) | 6275767524 | 6169882392 (98.31%) | 5956702080 (94.92%) | 90132 (0.00%) | 2669228466 (42.53%) |
| W22 | 6491621420 | 6368359301 (98.10%) | 6141621662 (94.61%) | 87914 (0.00%) | 2750269694 (42.37%) | 6449265199 | 6339048578 (98.29%) | 6115980017 (94.83%) | 87291 (0.00%) | 2728631330 (42.31%) |
| W23 | 6538588476 | 6414554223 (98.10%) | 6188282864 (94.64%) | 89730 (0.00%) | 2762907607 (42.26%) | 6491077050 | 6381481387 (98.31%) | 6159295327 (94.89%) | 88995 (0.00%) | 2738455113 (42.19%) |
| W31 | 6558460898 | 6432427598 (98.08%) | 6207703193 (94.65%) | 92480 (0.00%) | 2784775488 (42.46%) | 6509887160 | 6399116730 (98.30%) | 6178724919 (94.91%) | 91745 (0.00%) | 2759829867 (42.39%) |
| W32 | 6546432892 | 6420634996 (98.08%) | 6200707722 (94.72%) | 88165 (0.00%) | 2741330294 (41.88%) | 6491376982 | 6382803738 (98.33%) | 6167616092 (95.01%) | 87406 (0.00%) | 2713733342 (41.81%) |
| W33 | 6136160676 | 6014666312 (98.02%) | 5790773455 (94.37%) | 87235 (0.00%) | 2589423335 (42.20%) | 6096634323 | 5987245354 (98.21%) | 5766959091 (94.59%) | 86645 (0.00%) | 2569326540 (42.14%) |

Note: G01-03, G11-13, G21-23 and G31-33 represent three samples of Gannong NO. 3 at 0, 0.5, 1 and 2 h, respectively. W01-03, W11-13, W21-23, W31-33 represent three samples of ‘WL326GZ’ at 0, 0.5, 1 and 2 h, respectively.

Table S2 Results of mapping to the ribosome RNA (rRNA) database.

| Sample | clean_reads | Mapped_Reads (%) | Unmapped_Reads (%) |
| --- | --- | --- | --- |
| G01 | 43756714 | 2415274 (5.52%) | 41341440 (94.48%) |
| G02 | 43231954 | 718944 (1.66%) | 42513010 (98.34%) |
| G03 | 43731870 | 660022 (1.51%) | 43071848 (98.49%) |
| G11 | 39464248 | 494358 (1.25%) | 38969890 (98.75%) |
| G12 | 39642356 | 2971240 (7.50%) | 36671116 (92.50%) |
| G13 | 44495930 | 219898 (0.49%) | 44276032 (99.51%) |
| G21 | 41370718 | 141568 (0.34%) | 41229150 (99.66%) |
| G22 | 40647542 | 153710 (0.38%) | 40493832 (99.62%) |
| G23 | 44466474 | 2758376 (6.20%) | 41708098 (93.80%) |
| G31 | 44401086 | 1543680 (3.48%) | 42857406 (96.52%) |
| G32 | 43888338 | 605982 (1.38%) | 43282356 (98.62%) |
| G33 | 43147616 | 337598 (0.78%) | 42810018 (99.22%) |
| W01 | 43568822 | 180224 (0.41%) | 43388598 (99.59%) |
| W02 | 44843886 | 418726 (0.93%) | 44425160 (99.07%) |
| W03 | 39371250 | 134858 (0.34%) | 39236392 (99.66%) |
| W11 | 44211166 | 120232 (0.27%) | 44090934 (99.73%) |
| W12 | 45466356 | 171930 (0.38%) | 45294426 (99.62%) |
| W13 | 44340578 | 988098 (2.23%) | 43352480 (97.77%) |
| W21 | 42023452 | 412382 (0.98%) | 41611070 (99.02%) |
| W22 | 43317588 | 190292 (0.44%) | 43127296 (99.56%) |
| W23 | 43603910 | 275488 (0.63%) | 43328422 (99.37%) |
| W31 | 43595724 | 528080 (1.21%) | 43067644 (98.79%) |
| W32 | 43535050 | 331200 (0.76%) | 43203850 (99.24%) |
| W33 | 40815054 | 142726 (0.35%) | 40672328 (99.65%) |

Table S3 Results of clean data mapping to the alfalfa reference genome.

| Sample | Total | Unmapped (%) | Unique_Mapped (%) | Multiple_Mapped (%) | Total_Mapped (%) |
| --- | --- | --- | --- | --- | --- |
| G01 | 41341440 | 2816012 (6.81%) | 16905528 (40.89%) | 21619900 (52.30%) | 38525428 (93.19%) |
| G02 | 42513010 | 2659280 (6.26%) | 17655024 (41.53%) | 22198706 (52.22%) | 39853730 (93.74%) |
| G03 | 43071848 | 2757451 (6.40%) | 18040999 (41.89%) | 22273398 (51.71%) | 40314397 (93.60%) |
| G11 | 38969890 | 2637650 (6.77%) | 16218205 (41.62%) | 20114035 (51.61%) | 36332240 (93.23%) |
| G12 | 36671116 | 4017647 (10.96%) | 14584019 (39.77%) | 18069450 (49.27%) | 32653469 (89.04%) |
| G13 | 44276032 | 2886447 (6.52%) | 18521158 (41.83%) | 22868427 (51.65%) | 41389585 (93.48%) |
| G21 | 41229150 | 2836999 (6.88%) | 17424961 (42.26%) | 20967190 (50.86%) | 38392151 (93.12%) |
| G22 | 40493832 | 2989681 (7.38%) | 17006371 (42.00%) | 20497780 (50.62%) | 37504151 (92.62%) |
| G23 | 41708098 | 3227978 (7.74%) | 17047851 (40.87%) | 21432269 (51.39%) | 38480120 (92.26%) |
| G31 | 42857406 | 2703389 (6.31%) | 17445907 (40.71%) | 22708110 (52.99%) | 40154017 (93.69%) |
| G32 | 43282356 | 2595067 (6.00%) | 17922508 (41.41%) | 22764781 (52.60%) | 40687289 (94.00%) |
| G33 | 42810018 | 2993555 (6.99%) | 17179605 (40.13%) | 22636858 (52.88%) | 39816463 (93.01%) |
| W01 | 43388598 | 2872309 (6.62%) | 17956721 (41.39%) | 22559568 (51.99%) | 40516289 (93.38%) |
| W02 | 44425160 | 2986435 (6.72%) | 18699023 (42.09%) | 22739702 (51.19%) | 41438725 (93.28%) |
| W03 | 39236392 | 2565703 (6.54%) | 16664031 (42.47%) | 20006658 (50.99%) | 36670689 (93.46%) |
| W11 | 44090934 | 3064783 (6.95%) | 18412656 (41.76%) | 22613495 (51.29%) | 41026151 (93.05%) |
| W12 | 45294426 | 2888769 (6.38%) | 19372132 (42.77%) | 23033525 (50.85%) | 42405657 (93.62%) |
| W13 | 43352480 | 3026074 (6.98%) | 18177904 (41.93%) | 22148502 (51.09%) | 40326406 (93.02%) |
| W21 | 41611070 | 2583532 (6.21%) | 17805373 (42.79%) | 21222165 (51.00%) | 39027538 (93.79%) |
| W22 | 43127296 | 2870120 (6.65%) | 18221205 (42.25%) | 22035971 (51.10%) | 40257176 (93.35%) |
| W23 | 43328422 | 2898426 (6.69%) | 18259085 (42.14%) | 22170911 (51.17%) | 40429996 (93.31%) |
| W31 | 43067644 | 2719727 (6.32%) | 18497940 (42.95%) | 21849977 (50.73%) | 40347917 (93.68%) |
| W32 | 43203850 | 3109980 (7.20%) | 18865121 (43.67%) | 21228749 (49.14%) | 40093870 (92.80%) |
| W33 | 40672328 | 2712450 (6.67%) | 17270647 (42.46%) | 20689231 (50.87%) | 37959878 (93.33%) |

Table S4 All genes generated in the samples by RNA-seq.

| sample | Refer_Genes | sequenced_Refer_Genes (%) | Novel_Genes | sequenced_Novel_Genes (%) | Total_Genes | sequenced_Total_Genes (%) |
| --- | --- | --- | --- | --- | --- | --- |
| G01 | 164632 | 65852 (40.00%) | 9680 | 7267 (75.07%) | 174312 | 73119 (41.95%) |
| G02 | 164632 | 69425 (42.17%) | 9680 | 7575 (78.25%) | 174312 | 77000 (44.17%) |
| G03 | 164632 | 69652 (42.31%) | 9680 | 7597 (78.48%) | 174312 | 77249 (44.32%) |
| G11 | 164632 | 69098 (41.97%) | 9680 | 7550 (78.00%) | 174312 | 76648 (43.97%) |
| G12 | 164632 | 68375 (41.53%) | 9680 | 7434 (76.80%) | 174312 | 75809 (43.49%) |
| G13 | 164632 | 72430 (44.00%) | 9680 | 7852 (81.12%) | 174312 | 80282 (46.06%) |
| G21 | 164632 | 70969 (43.11%) | 9680 | 7539 (77.88%) | 174312 | 78508 (45.04%) |
| G22 | 164632 | 71362 (43.35%) | 9680 | 7660 (79.13%) | 174312 | 79022 (45.33%) |
| G23 | 164632 | 64744 (39.33%) | 9680 | 7142 (73.78%) | 174312 | 71886 (41.24%) |
| G31 | 164632 | 67181 (40.81%) | 9680 | 7376 (76.20%) | 174312 | 74557 (42.77%) |
| G32 | 164632 | 70075 (42.56%) | 9680 | 7583 (78.34%) | 174312 | 77658 (44.55%) |
| G33 | 164632 | 71612 (43.50%) | 9680 | 7807 (80.65%) | 174312 | 79419 (45.56%) |
| W01 | 164632 | 72185 (43.85%) | 9680 | 7798 (80.56%) | 174312 | 79983 (45.88%) |
| W02 | 164632 | 73287 (44.52%) | 9680 | 7853 (81.13%) | 174312 | 81140 (46.55%) |
| W03 | 164632 | 71285 (43.30%) | 9680 | 7642 (78.95%) | 174312 | 78927 (45.28%) |
| W11 | 164632 | 72850 (44.25%) | 9680 | 7846 (81.05%) | 174312 | 80696 (46.29%) |
| W12 | 164632 | 72284 (43.91%) | 9680 | 7736 (79.92%) | 174312 | 80020 (45.91%) |
| W13 | 164632 | 71269 (43.29%) | 9680 | 7700 (79.55%) | 174312 | 78969 (45.30%) |
| W21 | 164632 | 69461 (42.19%) | 9680 | 7506 (77.54%) | 174312 | 76967 (44.15%) |
| W22 | 164632 | 71399 (43.37%) | 9680 | 7738 (79.94%) | 174312 | 79137 (45.40%) |
| W23 | 164632 | 73734 (44.79%) | 9680 | 7761 (80.18%) | 174312 | 81495 (46.75%) |
| W31 | 164632 | 69475 (42.20%) | 9680 | 7440 (76.86%) | 174312 | 76915 (44.12%) |
| W32 | 164632 | 63234 (38.41%) | 9680 | 6970 (72.00%) | 174312 | 70204 (40.27%) |
| W33 | 164632 | 70809 (43.01%) | 9680 | 7584 (78.35%) | 174312 | 78393 (44.97%) |
| all | 164632 | 111686 (67.84%) | 9680 | 9680 (100.00%) | 174312 | 121366 (69.63%) |

Table S5 DEGs of ‘Gannong NO. 3’ in top 3 profiles enriched in the GO terms.

| profile 8 | | | | | |
| --- | --- | --- | --- | --- | --- |
| GO ID | Descrption | number of DEGs | percent (%) | P-value | Q-value |
| GO:0044699 | single-organism process | 571 | 81.34 | 3.54E-19 | 8.95E-17 |
| GO:0003824 | catalytic activity | 535 | 78.68 | 1.99E-11 | 1.22E-09 |
| GO:0044763 | single-organism cellular process | 424 | 60.4 | 6.59E-09 | 1.67E-07 |
| GO:0044710 | single-organism metabolic process | 411 | 58.55 | 8.24E-28 | 8.34E-25 |
| GO:0005737 | cytoplasm | 293 | 50.52 | 5.82E-06 | 6.50E-05 |
| GO:0050896 | response to stimulus | 350 | 49.86 | 5.62E-07 | 8.61E-06 |
| GO:0016020 | membrane | 275 | 47.41 | 1.33E-05 | 0.000137 |
| GO:0042221 | response to chemical | 254 | 36.18 | 4.37E-14 | 4.02E-12 |
| GO:0009536 | plastid | 198 | 34.14 | 4.10E-11 | 2.75E-09 |
| GO:0006950 | response to stress | 232 | 33.05 | 1.79E-08 | 3.95E-07 |
| GO:0044425 | membrane part | 175 | 30.17 | 1.87E-07 | 4.18E-06 |
| GO:0044281 | small molecule metabolic process | 202 | 28.78 | 3.01E-10 | 8.70E-09 |
| GO:0031224 | intrinsic component of membrane | 162 | 27.93 | 1.12E-07 | 3.01E-06 |
| GO:0016491 | oxidoreductase activity | 166 | 24.41 | 3.24E-16 | 4.97E-14 |
| GO:0043436 | oxoacid metabolic process | 165 | 23.5 | 6.94E-12 | 4.25E-10 |
| GO:0006082 | organic acid metabolic process | 165 | 23.5 | 7.14E-12 | 4.25E-10 |
| GO:0009628 | response to abiotic stimulus | 159 | 22.65 | 2.22E-06 | 3.04E-05 |
| GO:0019752 | carboxylic acid metabolic process | 156 | 22.22 | 6.02E-11 | 2.76E-09 |
| GO:0010033 | response to organic substance | 154 | 21.94 | 5.53E-06 | 7.09E-05 |
| GO:0044711 | single-organism biosynthetic process | 151 | 21.51 | 6.78E-12 | 4.25E-10 |
| profile 11 |  |  |  |  |  |
| GO ID | Descrption | number of DEGs | percent (%) | P-value | Q-value |
| GO:0050896 | response to stimulus | 400 | 61.73 | 2.55E-27 | 5.23E-25 |
| GO:0042221 | response to chemical | 270 | 41.67 | 1.76E-24 | 2.25E-22 |
| GO:0006950 | response to stress | 262 | 40.43 | 4.63E-21 | 3.96E-19 |
| GO:0050794 | regulation of cellular process | 218 | 33.64 | 3.18E-11 | 1.12E-09 |
| GO:0010033 | response to organic substance | 210 | 32.41 | 1.13E-26 | 1.94E-24 |
| GO:0009628 | response to abiotic stimulus | 177 | 27.32 | 1.37E-13 | 6.71E-12 |
| GO:0051716 | cellular response to stimulus | 177 | 27.32 | 1.17E-11 | 4.44E-10 |
| GO:0009719 | response to endogenous stimulus | 175 | 27.01 | 1.52E-31 | 3.90E-29 |
| GO:0009605 | response to external stimulus | 167 | 25.77 | 1.36E-18 | 1.07E-16 |
| GO:0007154 | cell communication | 161 | 24.85 | 6.92E-16 | 3.95E-14 |
| GO:0007165 | signal transduction | 146 | 22.53 | 6.53E-18 | 4.22E-16 |
| GO:0023052 | signaling | 146 | 22.53 | 6.99E-18 | 4.22E-16 |
| GO:0044700 | single organism signaling | 146 | 22.53 | 6.99E-18 | 4.22E-16 |
| GO:0051704 | multi-organism process | 143 | 22.07 | 1.55E-18 | 1.13E-16 |
| GO:0009607 | response to biotic stimulus | 142 | 21.91 | 3.54E-22 | 4.03E-20 |
| GO:0051707 | response to other organism | 138 | 21.3 | 1.14E-21 | 1.17E-19 |
| GO:0043207 | response to external biotic stimulus | 138 | 21.3 | 4.22E-21 | 3.93E-19 |
| GO:0001101 | response to acid chemical | 116 | 17.9 | 1.42E-12 | 5.83E-11 |
| GO:1901698 | response to nitrogen compound | 105 | 16.2 | 9.35E-39 | 4.80E-36 |
| GO:0010243 | response to organonitrogen compound | 103 | 15.9 | 7.79E-52 | 7.99E-49 |
| profile16 |  |  |  |  |  |
| GO ID | Descrption | number of DEGs | percent (%) | P-value | Q-value |
| GO:0005737 | cytoplasm | 132 | 56.9 | 1.45E-06 | 1.51E-05 |
| GO:0044444 | cytoplasmic part | 131 | 56.47 | 9.01E-07 | 1.01E-05 |
| GO:0009536 | plastid | 85 | 36.64 | 5.01E-07 | 6.76E-06 |
| GO:0044422 | organelle part | 85 | 36.64 | 4.40E-05 | 0.000349 |
| GO:0043167 | ion binding | 92 | 36.51 | 1.37E-06 | 0.000144 |
| GO:0043169 | cation binding | 88 | 34.92 | 6.27E-07 | 0.000132 |
| GO:0044446 | intracellular organelle part | 76 | 32.76 | 3.25E-06 | 3.13E-05 |
| GO:0044435 | plastid part | 53 | 22.85 | 6.91E-09 | 1.56E-07 |
| GO:0030312 | external encapsulating structure | 37 | 15.95 | 6.53E-07 | 8.02E-06 |
| GO:0071944 | cell periphery | 37 | 15.95 | 7.35E-06 | 6.20E-05 |
| GO:0044434 | chloroplast part | 30 | 12.93 | 1.29E-11 | 1.75E-09 |
| GO:0009507 | chloroplast | 30 | 12.93 | 3.56E-11 | 2.40E-09 |
| GO:0005576 | extracellular region | 30 | 12.93 | 1.62E-07 | 3.11E-06 |
| GO:0044283 | small molecule biosynthetic process | 31 | 10.99 | 2.73E-05 | 0.00144 |
| GO:0005618 | cell wall | 23 | 9.91 | 1.84E-07 | 3.11E-06 |
| GO:0016053 | organic acid biosynthetic process | 27 | 9.57 | 7.53E-06 | 0.000663 |
| GO:0046394 | carboxylic acid biosynthetic process | 27 | 9.57 | 7.53E-06 | 0.000663 |
| GO:0006790 | sulfur compound metabolic process | 27 | 9.57 | 3.02E-05 | 0.001494 |
| GO:0046906 | tetrapyrrole binding | 23 | 9.13 | 3.55E-06 | 0.00025 |
| GO:0044272 | sulfur compound biosynthetic process | 24 | 8.51 | 6.22E-06 | 0.000663 |

Note: Only showed the top 20 GO-terms of each profile in this table.

Table S6 DEGs of ‘WL326GZ’ in top 3 profiles enriched in the GO terms.

| profile 18 | | | | | |
| --- | --- | --- | --- | --- | --- |
| GO ID | Descrption | number of DEGs | percent (%) | P-value | Q-value |
| GO:0050896 | response to stimulus | 291 | 67.67 | 1.12E-29 | 8.34E-28 |
| GO:0065007 | biological regulation | 219 | 50.93 | 2.04E-10 | 3.70E-09 |
| GO:0042221 | response to chemical | 213 | 49.54 | 1.33E-31 | 1.10E-29 |
| GO:0050789 | regulation of biological process | 210 | 48.84 | 1.89E-11 | 3.90E-10 |
| GO:0006950 | response to stress | 197 | 45.81 | 1.58E-23 | 9.01E-22 |
| GO:0010033 | response to organic substance | 183 | 42.56 | 9.04E-41 | 1.68E-38 |
| GO:0050794 | regulation of cellular process | 156 | 36.28 | 4.13E-11 | 8.28E-10 |
| GO:0009719 | response to endogenous stimulus | 155 | 36.05 | 1.60E-44 | 3.95E-42 |
| GO:0051716 | cellular response to stimulus | 137 | 31.86 | 1.31E-14 | 3.03E-13 |
| GO:0009605 | response to external stimulus | 132 | 30.7 | 6.40E-22 | 3.39E-20 |
| GO:0007154 | cell communication | 131 | 30.47 | 6.28E-21 | 2.91E-19 |
| GO:0009628 | response to abiotic stimulus | 121 | 28.14 | 1.19E-10 | 2.21E-09 |
| GO:0007165 | signal transduction | 114 | 26.51 | 7.35E-20 | 2.76E-18 |
| GO:0023052 | signaling | 114 | 26.51 | 7.81E-20 | 2.76E-18 |
| GO:0044700 | single organism signaling | 114 | 26.51 | 7.81E-20 | 2.76E-18 |
| GO:0051704 | multi-organism process | 106 | 24.65 | 1.29E-17 | 3.42E-16 |
| GO:0043207 | response to external biotic stimulus | 105 | 24.42 | 6.10E-21 | 2.91E-19 |
| GO:0009607 | response to biotic stimulus | 105 | 24.42 | 2.23E-20 | 9.21E-19 |
| GO:0051707 | response to other organism | 103 | 23.95 | 2.23E-20 | 9.21E-19 |
| GO:1901698 | response to nitrogen compound | 98 | 22.79 | 9.47E-50 | 3.51E-47 |
| GO:0009725 | response to hormone | 98 | 22.79 | 7.52E-17 | 1.92E-15 |
| profile 11 |  |  |  |  |  |
| GO ID | Descrption | number of DEGs | percent (%) | P-value | Q-value |
| GO:0050896 | response to stimulus | 264 | 66.17 | 8.39E-25 | 1.33E-22 |
| GO:0065007 | biological regulation | 204 | 51.13 | 5.43E-10 | 1.02E-08 |
| GO:0050789 | regulation of biological process | 190 | 47.62 | 2.16E-09 | 3.64E-08 |
| GO:0009696 | salicylic acid metabolic process | 42 | 10.53 | 5.98E-25 | 1.18E-22 |
| GO:0042221 | response to chemical | 174 | 43.61 | 1.12E-18 | 4.90E-17 |
| GO:0006950 | response to stress | 173 | 43.36 | 7.08E-18 | 2.94E-16 |
| GO:0050794 | regulation of cellular process | 148 | 37.09 | 1.99E-11 | 4.92E-10 |
| GO:0010033 | response to organic substance | 139 | 34.84 | 2.47E-21 | 1.50E-19 |
| GO:0009719 | response to endogenous stimulus | 130 | 32.58 | 1.92E-32 | 5.05E-30 |
| GO:0007154 | cell communication | 122 | 30.58 | 9.64E-20 | 4.76E-18 |
| GO:0051716 | cellular response to stimulus | 122 | 30.58 | 7.82E-12 | 2.13E-10 |
| GO:0007165 | signal transduction | 112 | 28.07 | 1.23E-21 | 8.57E-20 |
| GO:0023052 | signaling | 112 | 28.07 | 1.30E-21 | 8.57E-20 |
| GO:0044700 | single organism signaling | 112 | 28.07 | 1.30E-21 | 8.57E-20 |
| GO:0009605 | response to external stimulus | 109 | 27.32 | 1.82E-14 | 6.26E-13 |
| GO:0006464 | cellular protein modification process | 105 | 26.32 | 4.58E-06 | 3.77E-05 |
| GO:0036211 | protein modification process | 105 | 26.32 | 5.74E-06 | 4.63E-05 |
| GO:0051707 | response to other organism | 84 | 21.05 | 1.65E-13 | 5.23E-12 |
| GO:0001101 | response to acid chemical | 84 | 21.05 | 2.86E-13 | 8.68E-12 |
| GO:0043207 | response to external biotic stimulus | 84 | 21.05 | 3.64E-13 | 1.06E-11 |
| profile 9 |  |  |  |  |  |
| GO ID | Descrption | number of DEGs | percent (%) | P-value | Q-value |
| GO:0071704 | organic substance metabolic process | 315 | 77.4 | 4.65E-06 | 0.000226 |
| GO:0044237 | cellular metabolic process | 307 | 75.43 | 1.62E-07 | 2.16E-05 |
| GO:0044763 | single-organism cellular process | 255 | 62.65 | 8.83E-08 | 1.55E-05 |
| GO:0005737 | cytoplasm | 188 | 53.56 | 2.95E-06 | 6.50E-05 |
| GO:0044444 | cytoplasmic part | 186 | 52.99 | 2.15E-06 | 5.83E-05 |
| GO:0044710 | single-organism metabolic process | 204 | 50.12 | 7.20E-07 | 6.41E-05 |
| GO:0009058 | biosynthetic process | 166 | 40.79 | 9.15E-06 | 0.000408 |
| GO:1901576 | organic substance biosynthetic process | 158 | 38.82 | 3.84E-06 | 0.000205 |
| GO:0009536 | plastid | 126 | 35.9 | 4.58E-09 | 3.52E-07 |
| GO:0044422 | organelle part | 124 | 35.33 | 7.64E-06 | 0.000118 |
| GO:0044281 | small molecule metabolic process | 133 | 32.68 | 4.62E-11 | 4.94E-08 |
| GO:0051234 | establishment of localization | 126 | 30.96 | 2.92E-05 | 0.000924 |
| GO:0044446 | intracellular organelle part | 106 | 30.2 | 3.47E-06 | 6.68E-05 |
| GO:0009628 | response to abiotic stimulus | 107 | 26.29 | 6.89E-08 | 1.47E-05 |
| GO:0006793 | phosphorus metabolic process | 103 | 25.31 | 2.94E-05 | 0.000924 |
| GO:0043436 | oxoacid metabolic process | 95 | 23.34 | 2.41E-07 | 2.62E-05 |
| GO:0006082 | organic acid metabolic process | 95 | 23.34 | 2.45E-07 | 2.62E-05 |
| GO:0019752 | carboxylic acid metabolic process | 93 | 22.85 | 1.02E-07 | 1.55E-05 |
| GO:0044435 | plastid part | 77 | 21.94 | 2.61E-11 | 4.03E-09 |
| GO:1901564 | organonitrogen compound metabolic process | 80 | 19.66 | 7.80E-07 | 6.41E-05 |

Table S7 Gene Ontology enrichment analysis of the two main modules.

| GO ID | Descrption | percent (%) | ratio | Pvalue | Qvalue |
| --- | --- | --- | --- | --- | --- |
| darkred module |  |  |  |  |  |
| GO:0044699 | single-organism process | 74.233 | 0.018 | 2.10E-10 | 1.12E-08 |
| GO:0044763 | single-organism cellular process | 58.819 | 0.019 | 1.58E-11 | 1.05E-09 |
| GO:0044710 | single-organism metabolic process | 48.236 | 0.02 | 9.77E-14 | 1.36E-11 |
| GO:0050896 | response to stimulus | 47.699 | 0.018 | 1.65E-07 | 3.75E-06 |
| GO:0044444 | cytoplasmic part | 51.41 | 0.022 | 2.49E-14 | 3.57E-13 |
| GO:0005737 | cytoplasm | 51.41 | 0.022 | 1.20E-12 | 1.61E-11 |
| GO:0016020 | membrane | 47.761 | 0.022 | 9.97E-11 | 1.05E-09 |
| GO:0044422 | organelle part | 35.406 | 0.025 | 1.25E-16 | 2.79E-15 |
| GO:0051179 | localization | 31.825 | 0.021 | 9.86E-13 | 9.14E-11 |
| GO:0051234 | establishment of localization | 31.058 | 0.022 | 8.22E-14 | 1.27E-11 |
| GO:0009536 | plastid | 32.09 | 0.025 | 1.88E-15 | 3.79E-14 |
| GO:0044281 | small molecule metabolic process | 29.525 | 0.024 | 3.72E-20 | 5.18E-17 |
| GO:0042221 | response to chemical | 28.911 | 0.019 | 5.35E-06 | 8.18E-05 |
| GO:0006810 | transport | 28.528 | 0.021 | 7.50E-11 | 4.35E-09 |
| GO:0044425 | membrane part | 29.27 | 0.025 | 1.21E-11 | 1.53E-10 |
| GO:0043167 | ion binding | 28.972 | 0.019 | 2.53E-06 | 0.000106 |
| GO:0044446 | intracellular organelle part | 27.944 | 0.025 | 1.51E-11 | 1.79E-10 |
| GO:1902578 | single-organism localization | 25.153 | 0.022 | 4.16E-12 | 3.04E-10 |
| GO:0044765 | single-organism transport | 25.077 | 0.023 | 3.17E-12 | 2.55E-10 |
| GO:0031224 | intrinsic component of membrane | 26.7 | 0.025 | 3.37E-11 | 3.76E-10 |
|  |  |  |  |  |  |
| GO ID | Descrption | percent (%) | ratio | Pvalue | Qvalue |
| midnightblue module |  |  |  |  |  |
| GO:0005737 | cytoplasm | 62.5 | 0.004 | 3.19E-09 | 8.62E-08 |
| GO:0044444 | cytoplasmic part | 61.979 | 0.004 | 2.20E-09 | 7.43E-08 |
| GO:0044710 | single-organism metabolic process | 54.884 | 0.004 | 5.56E-07 | 2.42E-05 |
| GO:0044281 | small molecule metabolic process | 37.674 | 0.005 | 1.55E-10 | 1.28E-07 |
| GO:0009536 | plastid | 41.146 | 0.005 | 3.86E-09 | 8.68E-08 |
| GO:0044446 | intracellular organelle part | 35.417 | 0.005 | 4.64E-07 | 6.27E-06 |
| GO:0043436 | oxoacid metabolic process | 29.302 | 0.005 | 4.27E-09 | 5.49E-07 |
| GO:0006082 | organic acid metabolic process | 29.302 | 0.005 | 4.32E-09 | 5.49E-07 |
| GO:0019752 | carboxylic acid metabolic process | 28.372 | 0.006 | 4.65E-09 | 5.49E-07 |
| GO:0044711 | single-organism biosynthetic process | 24.186 | 0.005 | 1.36E-06 | 4.67E-05 |
| GO:0044435 | plastid part | 25.521 | 0.007 | 4.95E-10 | 2.23E-08 |
| GO:0032787 | monocarboxylic acid metabolic process | 22.326 | 0.006 | 5.71E-09 | 5.89E-07 |
| GO:0031967 | organelle envelope | 18.229 | 0.007 | 3.06E-07 | 4.59E-06 |
| GO:0031975 | envelope | 18.229 | 0.007 | 3.06E-07 | 4.59E-06 |
| GO:0019637 | organophosphate metabolic process | 14.419 | 0.006 | 3.74E-06 | 9.84E-05 |
| GO:0051186 | cofactor metabolic process | 13.953 | 0.009 | 1.46E-09 | 4.47E-07 |
| GO:0009526 | plastid envelope | 15.625 | 0.008 | 1.19E-07 | 2.29E-06 |
| GO:0044283 | small molecule biosynthetic process | 13.488 | 0.007 | 8.98E-07 | 3.23E-05 |
| GO:0019748 | secondary metabolic process | 12.558 | 0.009 | 6.67E-08 | 4.96E-06 |
| GO:0006790 | sulfur compound metabolic process | 12.558 | 0.008 | 1.73E-07 | 9.51E-06 |
